# FOXA1 is a transcriptional activator of *Odf2/Cenexin* and regulates primary ciliation

**DOI:** 10.1038/s41598-022-25966-w

**Published:** 2022-12-12

**Authors:** Christian Carl Czerny, Anett Borschel, Mingfang Cai, Madeline Otto, Sigrid Hoyer-Fender

**Affiliations:** 1 Johann-Friedrich-Blumenbach-Institute of Zoology and Anthropology – Developmental Biology, GZMB, Ernst-Caspari-Haus, Georg-August-Universität, Justus-von-Liebig-Weg 11, Göttingen, Germany; 2 Present Address: Thermo Fisher Scientific GENEART, Regensburg, Germany

**Keywords:** Cell biology, Molecular biology

## Abstract

Primary cilia are sensory organelles essential for embryonic and postnatal development, and tissue homeostasis in adulthood. They are generated in a cell cycle-dependent manner and found on most cells of the body. Although cilia formation is intensively investigated virtually nothing is known about the transcriptional regulation of primary ciliation. We used here *Odf2/Cenexin,* encoding a protein of the mother centriole and the basal body that is mandatory for primary cilia formation, as the target gene for the identification of transcriptional activators. We identified a consensus binding site for Fox transcription factors (TFs) in its promoter region and focused here on the Fox family. We found transcriptional activation of *Odf2* neither by FOXO TFs nor by the core TF for multiciliation, FOXJ1. However, we identified FOXA1 as a transcriptional activator of *Odf2* by reporter gene assays and qRT-PCR, and showed by qWB that *Foxa1* knockdown caused a decrease in ODF2 and CP110 proteins. We verified the binding sequence of FOXA1 in the *Odf2* promoter by ChIP. Finally, we demonstrated that knockdown of FOXA1 affected primary cilia formation. We, thus, showed for the first time, that FOXA1 regulates primary ciliation by transcriptional activation of ciliary genes.

## Introduction

Cilia are microtubule-based structures protruding from the cell surface into the environment. They are assembled at the distal part of the basal body, which is the former mother centriole. Basal bodies are attached to the cell membrane via their distal appendages thus exposing the ciliary axoneme outward. The core axonemal structure of all eukaryotic cilia and flagella consists of nine doublet microtubules arranged in a ring. This organization is known as a 9 + 0 structure and is found in immotile cilia^1^. The presence of two additional singlet microtubules and further accessory structures, such as e.g., dynein arms, characterizes motile cilia that are present in multitudes on the surface of specialized epithelial cells, such as those in the airway, the brain ventricles, and oviducts. Their coordinated movement enables the transport of fluids or cargos across the cell surface. In contrast, immotile cilia are present only once and are hence named primary or mono-cilia. Furthermore, primary cilia are found on the surface of nearly all cells of the body^2,3^. Primary cilia are essential sensory organelles, acting as mechano- and chemo-sensors and transducing signals in response to fluid flow in kidneys, mechanical load in bone, or ligand-activated receptors. Primary cilia are thus crucial for embryonic and postnatal development, and tissue homeostasis in adulthood. Dysfunctional primary cilia are causative for a large number of severe heritable diseases and syndromes, collectively classified as ciliopathies, as kidney and liver diseases, neural tube defects, and defects in left–right asymmetry^4^.

The generation of primary cilia is coordinated with the cell cycle and, vice versa, primary cilia might also affect the progression of the cell cycle and cell proliferation^5,6^. The formation of primary cilia starts in the G1-phase of the cell cycle and they are disassembled before mitosis^7^. However, primary cilia are predominantly found in cell-cycle arrested cells that have withdrawn from the cell cycle in the early G1 phase and entered a quiescent state (G0)^5,6,8,9^. In differentiated cells, quiescence is the terminal stage. However, when reversible, cells can be induced to re-enter the cell cycle in response to growth signals. Reversible quiescence characterises tissue-resident stem cells that function in replenishing tissue loss throughout life but is also found in non-stem cells, e.g. in endothelial cells^10^. Extrinsic and intrinsic factors regulate the cell cycle culminating in the activation of cyclin-dependent kinases, CDKs, by the binding to their regulatory cyclins that together drive the cell through the cell cycle^11–14^. The differential expression of cyclins throughout the cell cycle is mandatory for cell cycle progression^15,16^. Furthermore, cyclin-dependent kinase inhibitors, CKIs, antagonize cell cycle progression^14^. Extrinsic factors, as nutrients and growth factors, activate the mitogen-induced signaling pathway (MAPK pathway) and the serine/threonine-protein kinase B (PI3K/AKT) pathway eventually inducing transcription of cyclin D that binds and activates CDK4/6 thus initiating progression from G1 to S phase and cell proliferation^14^. On the other hand, low nutrient levels favour cell cycle arrest by increased AMP/ATP and ADP/ATP ratios which activate the energy-sensing AMP-activated protein kinase, AMPK. In between genes activated by AMPK is FOXO3a (also named FOXO3 or FOXO2)^17^. FOXO proteins are regulators of the cell cycle, metabolism, and apoptosis, and are inhibited by AKT-mediated phosphorylation^18–20^. Notably, FOXO3a inhibits mitochondrial gene expression, activates transcription of antioxidant enzymes, and regulates cell cycle progression^21–24^. Activation of FOXO3a, thus, correlates with cell cycle arrest and the formation of primary cilia.

The FOXO proteins belong to the large family of forkhead transcription factors (TFs) characterized by a winged-helix DNA-binding domain of ~ 110 amino acids (aa), termed the ‘forkhead box’ serving as the eponym for the whole Fox family^25–27^. The founding member *fork head* (*fkh*) was first identified in *Drosophila* as a region-specific homeotic gene required for the formation of terminal embryonic structures^28,29^. Soon thereafter, related genes were identified in diverse organisms ranging from yeast to humans. FOX TFs are evolutionary conserved and are required for a wide variety of biological functions in development and differentiation, and tissue homeostasis^30^. They have been categorized by sequence similarities into subclasses A to S^31^. The hepatocyte nuclear factor 3 forkhead homolog 4, HFH4, now renamed into FOXJ1, is essential for the formation of motile cilia. Homozygous *Foxj1*-deficient mice displayed randomized left–right asymmetry and a complete loss of motile multi-cilia in epithelial cells whereas sensory mono-cilia were present^32,33^. FOXJ1 is an essential component of the transcription factor network controlling the formation of motile cilia by regulating the expression of essential ciliary genes^34–37^. However, while a substantial insight into the transcriptional regulation of motile cilia has already been accumulated our knowledge about the transcriptional regulation of sensory mono-cilia is inconsiderable. FOX TFs are essential for diverse biological functions, including cell cycle regulation, thus raising the question if they are also involved in primary cilia formation. A first hint came from the ChIP-seq dataset from the ENCODE Transcription Factor Targets Datasets that lists several ciliary genes as FOXA1 target genes, including *Odf2*^38^. ODF2/Cenexin, which is a sub-distal appendage protein that marks the mother centriole and its derivative, the basal body, is mandatory for cilia formation^1,39–41^. *Odf2*-deficient cells miss the primary cilium and *Odf2*^*−/−*^ mice die during preimplantation^42,43^. Furthermore, the initiation of ciliogenesis depends on a crucial amount of ODF2 present at the mother centriole^44^. The tight regulation of *Odf2*, therefore, seems to be mandatory for the generation of primary cilia. We have carried out a candidate gene approach here, focusing on *Odf2,* which is essential for cilia formation, to investigate whether FOX TFs activate transcription of *Odf2* eventually promoting primary cilia formation. We have chosen the mouse fibroblast cell line NIH3T3 as an established cellular model for the investigation of primary cilia. Our results show that FOXA1 binds to the *Odf2* promoter and activates transcription to eventually regulate primary ciliogenesis.

## Results

### ODF2 is essential for primary cilia formation

**Figure 1:**
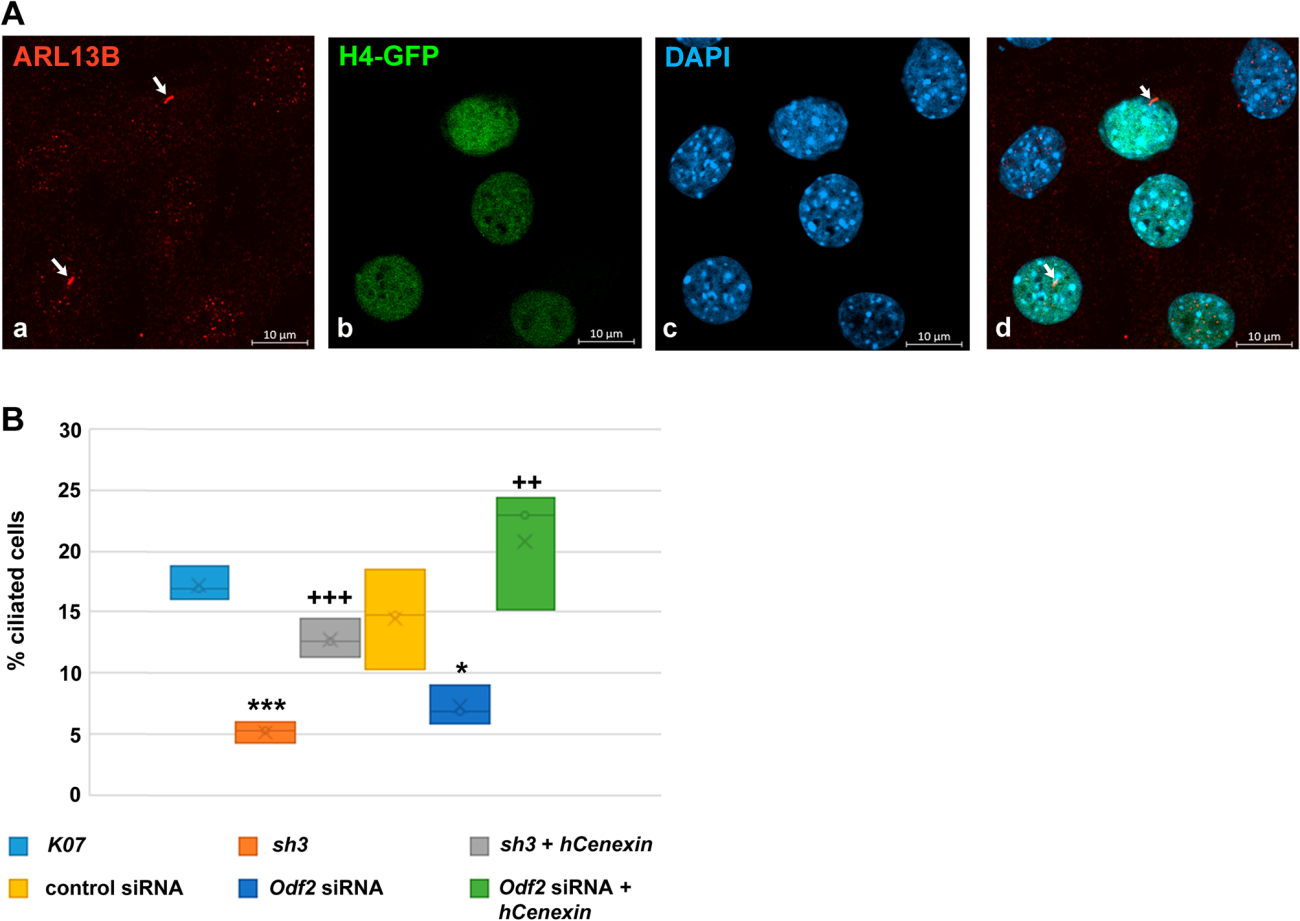
ODF2 is essential for primary cilia formation. (**A**) Exemplary presentation of ciliated NIH3T3 cells. Only transfected cells, whose nuclei stained green because of histone H4::GFP expression, were considered for ciliary counting. Cells were transfected with histone *H4::gfp* (**b**, green) and decorated for primary cilia with anti-ARL13B staining (**a**, red, arrows). Nuclei were stained with DAPI (**c**, blue), and the merged image is shown in d (cilia are marked by arrows). Scale bars in a–d of 10 µm. (**B**) The percentage of transfected cells that are ciliated is strongly decreased when *Odf2* was knocked down by transfection of either the short hairpin plasmid *sh3* or *Odf2* siRNA compared to the controls, plasmid *K07* or control siRNA, respectively. Co-transfection of the non-targeted *hCenexin* with either *sh3* or *Odf2* siRNA rescued primary cilia formation. Student’s T-test (one-tailed, homoscedastic): *p* < 0.05 * or ^+^, *p* < 0.01 ** or ^++^, *p* < 0.001 *** or ^+++^. Symbols represent comparison to either the respective controls (*K07* or control siRNA) * or to the respective knockdown (*sh3* or *Odf2* siRNA) ^+^. Biological triplicates with a total of n counted cells: *K07* n = 1,621, *sh3* n = 1,591, *sh3* + *hCenexin* n = 1,593, control siRNA n = 1,509, *Odf2* siRNA n = 1,538, *Odf2* siRNA + *hCenexi*n n = 1,580. *P*-values: *sh3* to *K07*: *p* = 0.000114***, *sh3* + *hCenexin* to *sh3* *p* = 0.000989^+++^, *Odf2* siRNA to control siRNA *p* = 0.022857*, and *Odf2* siRNA + *hCenexin* (rescue) to *Odf2* siRNA *p* = 0.005358^++^.

To validate the relevance of ODF2 for the formation of primary cilia, *Odf2* knockdown via transfection with either a short-hairpin plasmid (*sh3*) or siRNA was performed^45^. The plasmid *K07* or the scrambled non-target siRNA served as controls for *sh3*- or siRNA-mediated knockdown, respectively. For rescue, the human *Cenexin* plasmid (*hCenexin*^46^), was co-transfected with either *sh3* or *Odf2* siRNA. Additionally, to identify transfected cells, the plasmid encoding histone *H4* fused to *Egfp (H4::GFP)* was always co-transfected. Cells were fixed 24 h post-transfection and primary cilia immunologically decorated for the ciliary marker ARL13B (Fig. 1A). Primary cilia were manually counted by visual inspection and scanning through all focal planes taking into consideration only H4::GFP positive cells. We found a clear reduction in primary cilia when *Odf2* was knocked down by either the *sh3*-plasmid or siRNA when compared to the controls (*K07*-plasmid or scrambled non-target siRNA, respectively) (Fig. 1B). We have counted ~ 17% primary cilia in *K07*-transfected cells compared to only ~ 5% in *sh3*-transfected cells (*p* = 0.000114 ***), and ~ 15% in the siRNA control cells versus 7% in *Odf2* siRNA transfected cells (*p* = 0.022857 *). In contrast, the rescue experiment caused an increase in primary cilia to ~ 13% (*sh3* + *hCenexin*, *p* = 0.000989 ^+++^ to *sh3*), and ~ 21% (*Odf2* siRNA + *hCenexin*, *p* = 0.005358 ^++^ to *Odf2* siRNA). Our results, thus, confirmed ODF2 as being essential for primary cilia formation.

### FOXA1 is a transcriptional activator of the *Odf2* promoter

**Figure 2:**
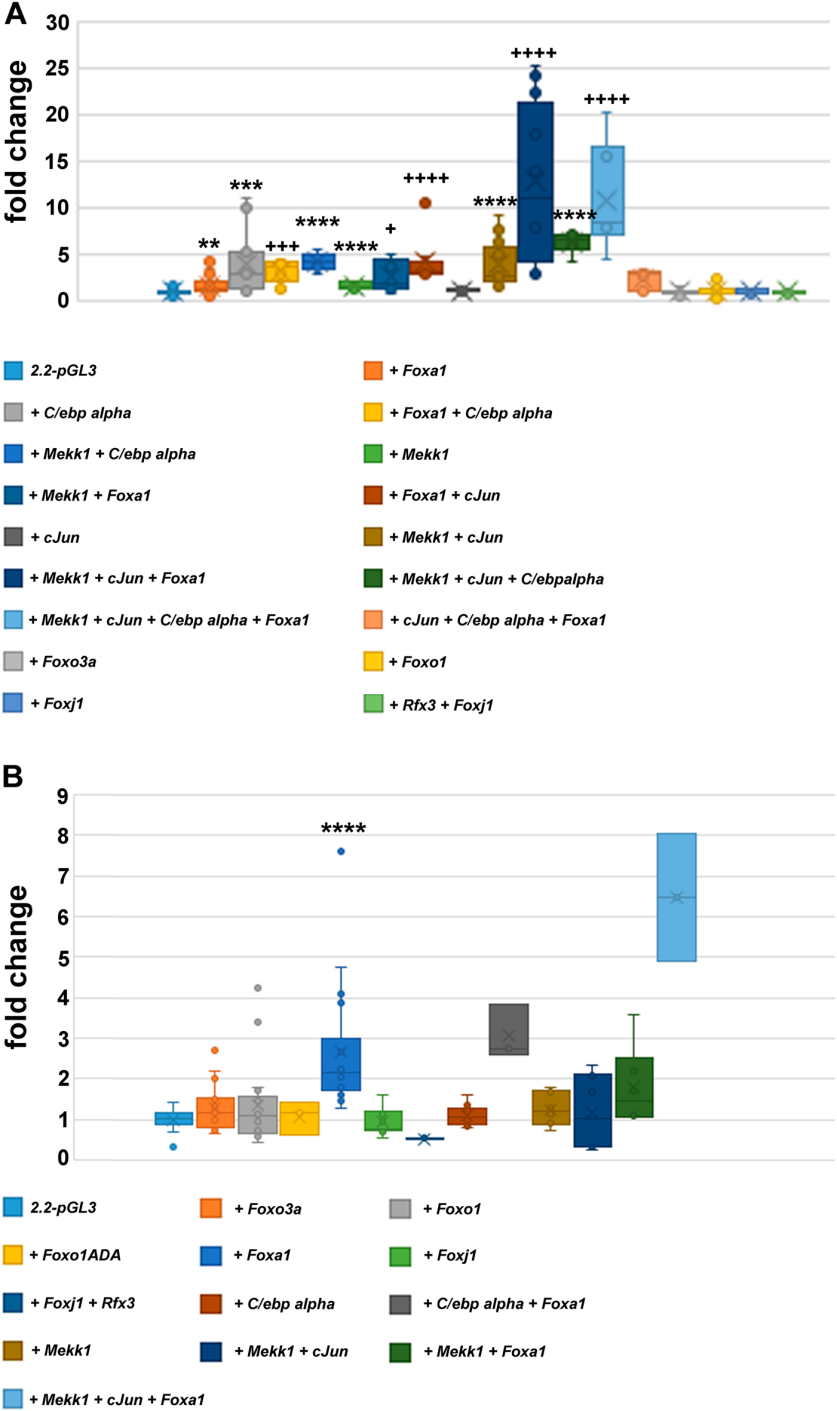
FOXA1 is a transcriptional activator of the *Odf2*-promoter. Co-expression of transcriptional activators, FOXO1, FOXO3A, FOXA1, FOXJ1, RFX3, C/EBPα, MEKK1, or cJUN with the firefly luciferase reporter under control of the *Odf2* promoter, *2.2-pGL3*, indicated FOXA1 as a transcriptional activator in cycling cells (**A**) and serum-starved cells (**B**), and suggested interaction between FOXA1 and cJUN. The firefly luciferase activity was related to the Renilla luciferase activity (encoded by *phRL*) as internal control and the fold changes of activity were calculated using the average of the relative luminescence of the control (*2.2-pGL3/phRL*) as 100% (or 1). Measurements of reporter gene activities were always performed in biological triplicates per experiment using a total of n biological replicates in cycling cells: n = 26 (control, *2.2-pGL3*), n = 24 (FOXO3A), n = 33 (FOXO1), n = 36 (FOXA1), n = 9 (FOXJ1), n = 6 (FOXJ1 + RFX3), n = 15 (C/EBPα), n = 9 (C/EBPα + FOXA1), n = 6 (C/EBPα + MEKK1), n = 9 (MEKK1), n = 9 (MEKK1 + FOXA1), n = 9 (FOXA1 + cJUN), n = 6 (cJUN), n = 18 (cJUN + MEKK1), n = 12 (cJUN + MEKK1 + FOXA1), n = 6 (MEKK1 + cJUN + C/EBPα), n = 6 (MEKK1 + cJUN + C/EBPα + FOXA1), n = 6 (cJUN + C/EBPα + FOXA1), and in serum-starved cells: n = 18 (control, *2.2-pGL3*), n = 18 (FOXO3A), n = 18 (FOXO1), n = 3 (FOXO1ADA), n = 18 (FOXA1), n = 9 (FOXJ1), n = 3 (FOXJ1 + RFX3), n = 9 (C/EBPα), n = 3 (C/EBPα + FOXA1), n = 6 (MEKK1), n = 6 (MEKK1 + FOXA1), n = 6 (MEKK1 + cJUN), n = 3 (cJUN + MEKK1 + FOXA1). Student’s T-test two-tailed, homoscedastic *p* < 0.05 *, *p* < 0.01 **, *p* < 0.001 ***, *p* < 0.0001 ****. Calculation of T-test to the control (*2.2-pGL3*/*phRL*) * or to *Foxa1* co-transfection ^**+**^.

We previously reported the characterization of the mouse *Odf2* promoter and identified C/EBPα and the stress-activated JNK-pathway as transcriptional activators^47^. Here, we identified a putative binding site for the forkhead-box TFs at position − 1775 of the *Odf2* promoter resembling the consensus binding sequence for FOXO (TT[G/A]TTTAC or GTAAA(T/C)AA;^48^), or FOXA1 (T(G/A)TT(T/G)AC;^49^). To verify whether TFs of the FOX-family are involved in transcriptional regulation of ciliary genes and formation of primary cilia, we first investigated the impact of forkhead-box TFs on the activation of the *Odf2* promoter, which controls expression of the firefly luciferase reporter gene (*2.2-pGL3*) ^47^. The reporter vector 2*.2-pGL3* comprised 1.8 kb upstream of the transcriptional start site of the mouse *Odf2* gene together with 358 bp of the transcribed region (− 1805/ + 358) positioned upstream of the firefly luciferase coding sequence. The reporter vector *2.2-pGL3* was co-transfected with the internal control vector *phRL-SV40,* which strongly expresses the Renilla luciferase under the SV40 promoter, and either without co-expression of TFs in the control probe or with, to investigate the effect of TFs on the activity of the *Odf2* promoter. The relative activity of the reporter vector was first calculated by dividing the light signals of the firefly luciferase by the light signals of the Renilla luciferase used as an internal control. The relative activity was then related to the average of the relative activity of the control probe, i.e. the reporter vector *2.2-pGL3* without co-expression of any TF, which was set to 1, finally giving the fold change in expression. When compared to the activity of the control probe, we observed a significant increase in reporter gene activity in cycling cells by *Foxa1* co-transfection (*p* = 0.001565**), but not by co-transfection of *Foxo1*, *Foxo3a*, *Foxj1*, or co-transfection of both, *Foxj1* and *Rfx3* (Fig. 2A). Additionally, our previous results reporting transcriptional activation by C/EBPα, and the JNK-pathway (MEKK1 + cJUN) were verified. The co-transfection of *Foxa1* with either *C/ebpα*, *Mekk1*, *cJun* or combinations of these factors revealed that FOXA1 together with C/EBPα has no additive effect on the reporter activity compared to C/EBPα. FOXA1 together with MEKK1 significantly increased the reporter gene activity compared to FOXA1 (*p* = 0.026646*), but without further increase when compared to MEKK1. FOXA1 together with cJUN increased the reporter gene activity to a similar extent as MEKK1-activated cJUN, and significantly (*p* = 2.84466 × 10^–5^****) when compared to FOXA1. The co-transfection of *Mekk1*, *cJun*, and *Foxa1* caused a further significant upregulation of the reporter activity compared to cJUN (*p* = 0.004115**), cJUN + MEKK1 (*p* = 0.0002307***), and FOXA1 (*p* = 3.37212 × 10^–10^****). These data indicated a positive interaction between FOXA1 and cJUN in the transcriptional activation of *Odf2* that is reinforced by MEKK1-mediated activation of cJUN. According to Student’s T-test, the co-expression of all four factors, MEKK1, cJUN, C/EBPα, and FOXA1 did not significantly increase the reporter gene activity compared to the co-expression of these factors but without FOXA1. The combination of FOXA1 with cJUN and C/EBPα did not cause an up-regulation of reporter gene activity as compared to FOXA1 alone.

Since cilia formation is stimulated by serum starvation-induced cellular quiescence simultaneously with enhanced *Odf2* promoter activity^47^ we wondered whether FOX TFs are also activated by serum starvation. The reporter gene experiment was therefore modified in that the medium was exchanged for serum-deprived medium 24 h post-transfection and cells cultivated for another 48 h before harvesting for luciferase assay. A significant increase in reporter gene activity was not observed for any of the transcription factors FOXO3A, FOXO1, FOXJ1, or FOXJ1 together with RFX3, or the constitutive active FOXO1, FOXO1ADA (Fig. 2B). In contrast, a significant increase was found for FOXA1 compared to the control (*2.2-pGL3* + *phRL*; *p* = 0.000041 ****). Increased reporter gene activity was also observed by co-expression of FOXA1 and C/EBPα when compared to the control (*2.2-pGL3* + *phRL*; *p* = 3.69035 × 10^–9^****), or to C/EBPα (*p* = 1.54407 × 10^–5^***) but not when compared to FOXA1. Significant activation of the reporter compared to the control (*2.2-pGL3* + *phRL*) was observed by co-expression of FOXA1 and MEKK1 (*p* = 0.003581**) but not when compared to FOXA1 indicating that MEKK1 did not affect FOXA1. Co-expression of MEKK1, cJUN, and FOXA1 caused a strong upregulation of the *Odf2* promoter compared to the control (*2.2-pGL3* + *phRL*; *p* = 3.18068 × 10^–12^****), to cJUN + MEKK1 (*p* = 0.0003696***), to MEKK1 + FOXA1 (*p* = 0.00084057***), or to FOXA1 (*p* = 0.0009507***) indicating an effect of MEKK1-activated cJUN and its putative positive interaction with FOXA1 on the activity of the *Odf2* promoter that was also observed in cycling cells (Fig. 2A).

**Figure 3:**
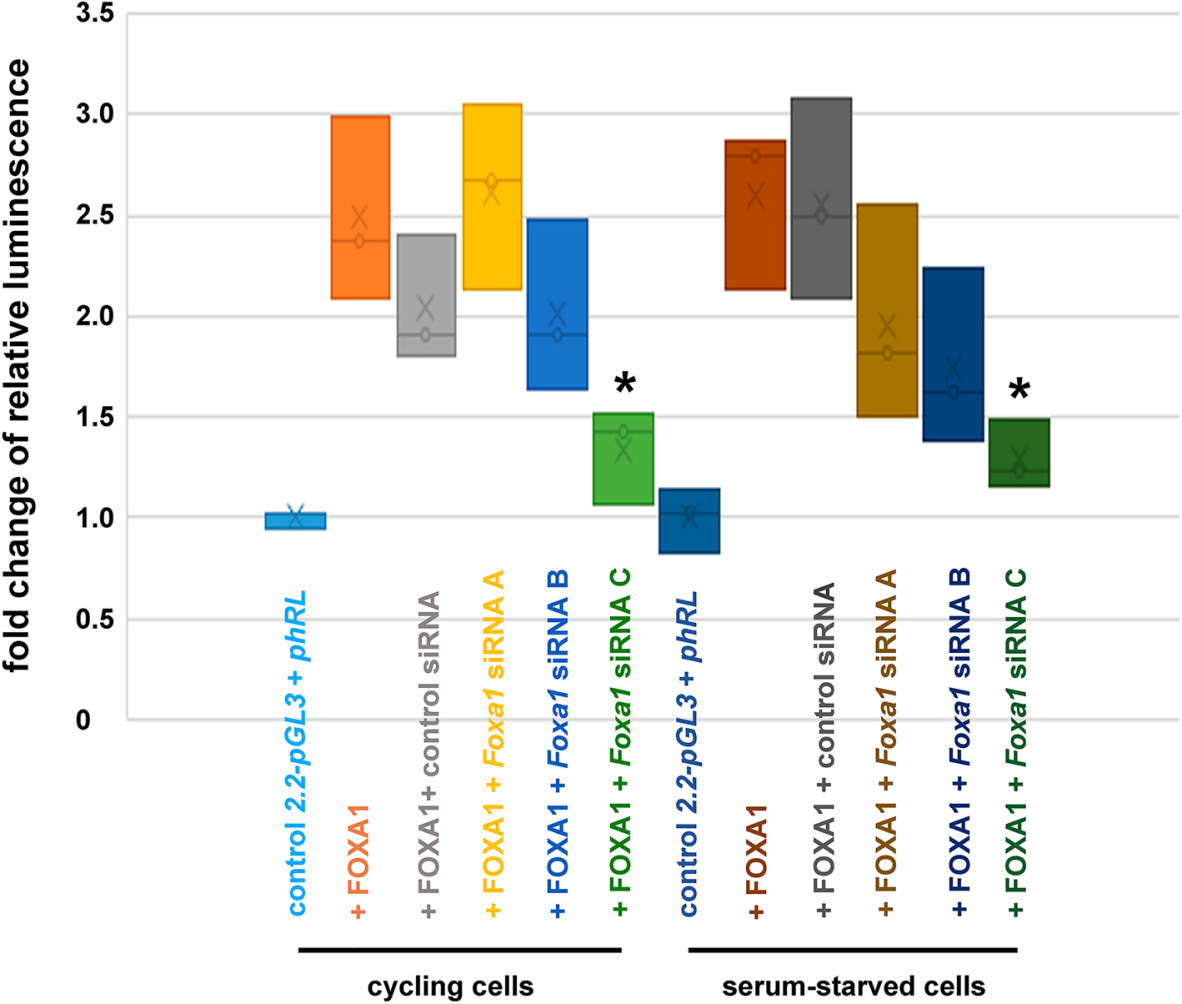
siRNA-mediated knockdown of *Foxa1* inhibits FOXA1-mediated activation of the *Odf2* promoter. Overexpression of FOXA1 (+ FOXA1) induced transcriptional upregulation of the firefly luciferase reporter controlled by the *Odf2* promoter (*2.2-pGL3*) compared to the control (*2.2-pGL3* + *phRL*) in both cycling cells and serum-starved cells. Co-transfection of the scrambled non-target siRNA (+ FOXA1 + control siRNA) did not significantly change the activity of the firefly luciferase. Co-transfection of either one of the three *Foxa1* siRNA duplexes (+ FOXA1 + *Foxa1* siRNA A, B, or C, respectively) repressed activation of the reporter vector in both cycling cells and serum-starved cells. Significant transcriptional repression by *Foxa1* siRNA C (in cycling cells: *p* = 0.039532*, in serum-starved cells: *p* = 0.014283*, Student’s T-test, two-tailed, homoscedastic). Three biological replicates each.

To further verify FOXA1 as a transcriptional activator of the *Odf2* promoter, the reporter vector 2*.2-pGL3* was co-transfected with the *Foxa1* expression plasmid, and either the negative control siRNA duplex, or one of the *Foxa1* siRNA duplexes A, B, or C. The activities of the firefly luciferase reporter and the *Renilla* luciferase as the internal control were measured either 24 h post-transfection (cycling cells) or after 48 h cultivation in serum starvation medium (cell cycle-arrested cells). Calculation of the relative luminescence revealed an approximately 2.5-fold increase of the reporter gene activity when expression of FOXA1 was enforced compared to control cells in which only the reporter *2.2-pGL3* and the internal control *phRL* were co-transfected (Fig. 3). Co-transfection of the negative control siRNA did not significantly change the expression of the reporter vector. However, we observed reduced activity of the reporter vector when *Foxa1* siRNA duplexes B or C were co-transfected while siRNA A was not effective. Though, significant reductions in reporter gene activity (p*) were obtained exclusively for the *Foxa1* siRNA duplex C in both, cycling cells (*p* = 0.03953205*) as well as in quiescent cells (*p* = 0.0142835*). Since the FOXA1-mediated activation of the *Odf2* promoter can be efficiently repressed by *Foxa1* siRNA duplexes these data demonstrate the importance of FOXA1 for the transcriptional activation of *Odf2*, as well as the specificity of *Foxa1* siRNA duplexes for the knockdown of FOXA1.

### FOXA1 binds to the *Odf2* promoter

**Figure 4:**
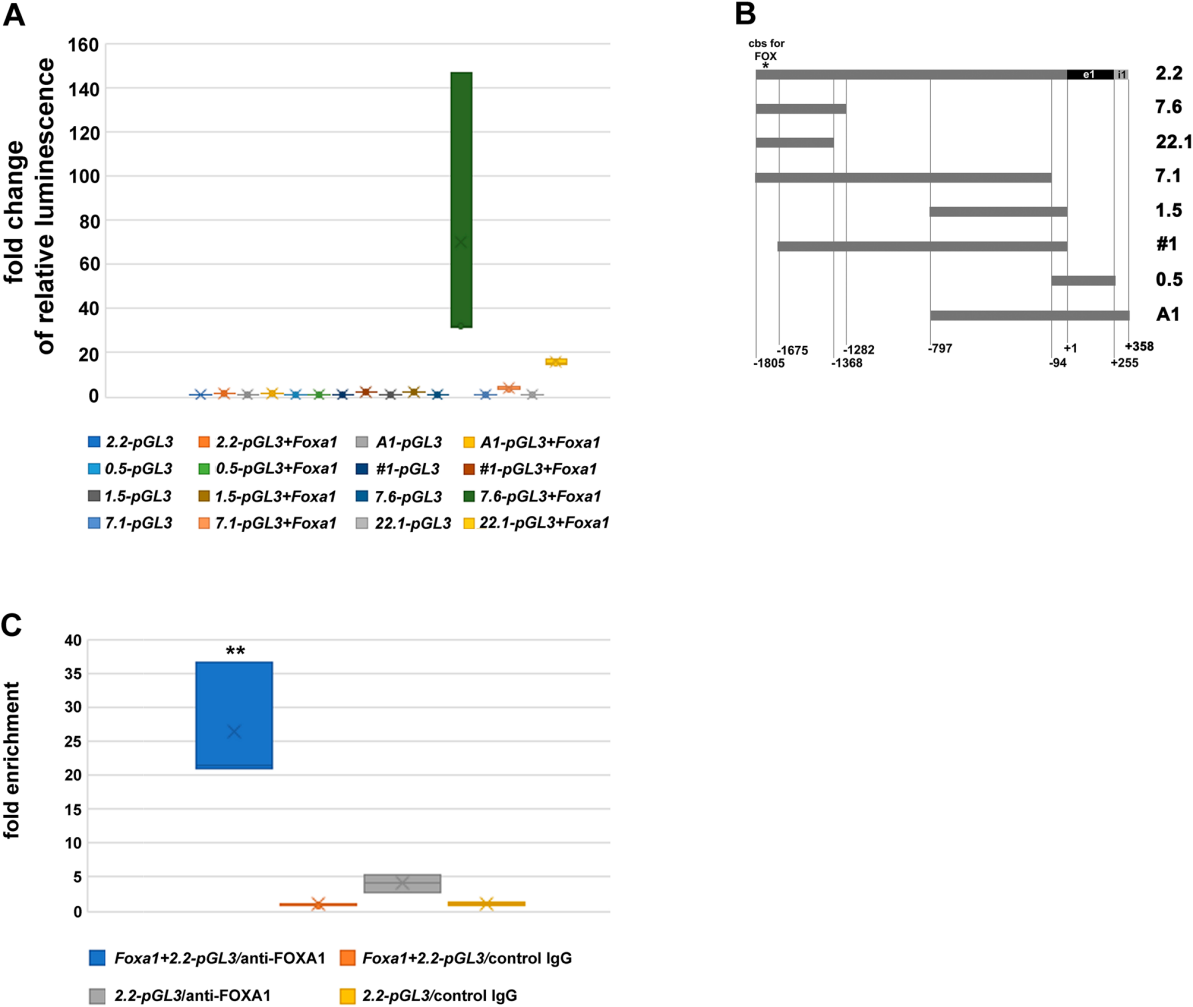
FOXA1 binds to the sequence TGTTTAC situated at positions -1768 to -1775 of the *Odf2* promoter. (**A**) Reporter gene assay to narrow down the FOXA1-binding site. Reporter vectors comprising specific parts of the *Odf2* promoter were investigated for their activation by FOXA1. FOXA1-mediated transcriptional activation was always related to that of the respective vector, without FOXA1 co-expression. The strongest activation was observed for the reporter vectors 7.6 (~ 70x), 22.1 (~ 16x), and 7.1 (~ 4x) indicating that the FOXA1-binding site is positioned in the region − 1282 to − 1805. Three biological replicates. (**B**) Scheme of the *Odf2* promoter fragments cloned upstream of the firefly reporter gene (e1 = exon1, i1 = intron1). The consensus binding site (cbs) for FOX TFs is located at position − 1768 to − 1775 (*). (**C**) Chromatin immune-precipitation by FOXA1 and qPCR of the binding-site sequence revealed significant enrichment. NIH3T3 cells were transfected with the *Odf2* promoter reporter vector *2.2-pGL3* as bait. Co-transfection of the *Foxa1* expression plasmid (*Foxa1* + *2.2-pGL3*/anti-FOXA1) resulted in an ~ 26 × enrichment of the FOXA1-binding site compared to the control IgGs (*Foxa1* + *2.2-pGL3*/control IgG) (p**, *p* = 0.007838). Precipitation of the endogenous FOXA1 by anti-FOXA1 antibodies (*2.2-pGL3*/anti-FOXA1) caused an ~ 4 × enrichment of the binding sequence compared to the control (*2.2-pGL3*/control IgG) that is, however, not significant (*p* = 0.137344).

Reporter gene assays were performed to narrow down the binding site of FOXA1 in the *Odf2* promoter. Fragments of the *Odf2* promoter, cloned upstream of the firefly reporter gene in *pGL3* as described in Pletz et al.^47^ (Fig. 4B), were investigated for their responsiveness to the FOXA1 transcription factor. Here, FOXA1-induced transcriptional activation was related to the basal level of the respective reporter construct without *Foxa1* co-transfection. Strong induction of transcription was observed for the promoter region − 1282 to − 1805 in clone *7.6* (~ 70 × when co-transfected with *Foxa1* as compared to the control without FOXA1 overexpression), for the region − 1368 to − 1805 in clone *22.1* (~ 16x), and the region − 94 to − 1805 in clone *7.1* (~ 4x) (Fig. 4 A), indicating that the FOXA1-binding site most likely is located in the region between − 1282 and − 1805 of the *Odf2*-promoter.

A sequence (TGTTTAC) with similarity to the FOXA1 consensus binding sequence T(G/A)TT(T/G)AC^49^ was identified in the *Odf2* promoter at position − 1768 to − 1775 upstream of the transcription start site. To investigate the binding of FOXA1 to this sequence we performed chromatin immune-precipitation using the *Odf2*-promoter reporter vector *2.2-pGL3* as bait and the anti-FOXA1 antibody as the fishing agent. Furthermore, ChIP was additionally performed in cells co-transfected with the *Foxa1* expression plasmid. Enrichment of the binding sequence was investigated by qPCR using primers that flank the supposed binding site and obtained Ct-values were adjusted to the input (ΔCt), and the control ΔCt-values (ΔΔCt). We found ~ 26 × enrichment of the binding site in cells co-transfected with the *Foxa1* expression plasmid (*p* < 0.01**), and ~ 4 × enrichment when the endogenous FOXA1 was precipitated (*p* = 0.137344, Student’s T-test two-tailed, homoscedastic) (Fig. 4C). No enrichment was found for the sequence of the putative DREAM binding site (not shown) that we identified at positions + 27 to + 33 related to the transcription start site indicating the specificity of FOXA1-binding. Although precipitation of the endogenous FOXA1 did not reveal significant enrichment of the target sequence compared to the control IgGs, co-expression of FOXA1 together with the *Odf2*-reporter *2.2-pGL3* as bait resulted in a significant enrichment of the target sequence. These results indicated that the binding site of FOXA1 in the *Odf2* promoter is represented by the sequence TGTTTAC situated at position − 1768 to − 1775 upstream of the transcription start site.

### Expression of Fox transcription factors in NIH3T3 cells

**Figure 5:**
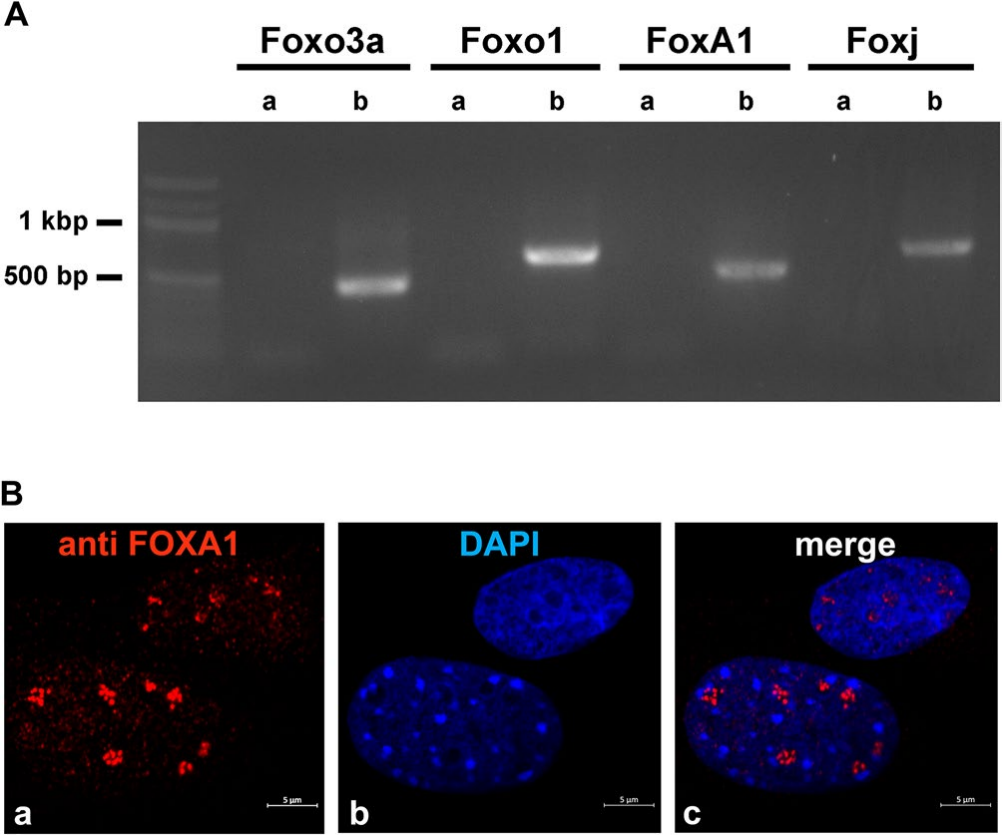
Expression of *Fox* TFs in NIH3T3 cells. (**A**) Amplification of *Fox* transcripts by first (**a**) and secondary/nested RT-PCR (**b**). Whereas in the first RT-PCR reaction no amplification products could be identified, the nested PCR performed on the first RT-PCR products as templates revealed products of the expected length of 449 bp (*Foxo3a*), 631 bp (*Foxo1*), 525 bp (*Foxa1*), and 674 bp (*Foxj1*). Correct amplification products were verified by sequencing. The original gel is presented in Supplementary Figure S3. (**B**) Immunological detection of endogenous FOXA1 in NIH3T3 nuclei (**a**, red), nuclear stain with DAPI (**b**, blue), and merge image (**c**). Bars are of 5 µm.

Our data indicated FOXA1 as an activator of *Odf2* transcription in NIH3T3 fibroblasts. However, it was unknown whether *Foxa1* or any other TF of the Fox-family is indeed expressed in NIH3T3 cells. We, therefore, verified the expression of *Fox* TFs by RT-PCR using cDNA prepared from NIH3T3 cells. Although no products were present after the first RT-PCR round, a nested PCR reaction using the first RT-PCR reaction as the template, revealed products of the expected lengths for all Fox TFs investigated (Fig. 5A). The correct amplifications were verified by sequencing PCR products. Thus, all four transcription factors investigated, *Foxa1*, *Foxo1*, *Foxo3a*, and *Foxj1,* were expressed in NIH3T3 cells albeit at a low level. Furthermore, the endogenous FOXA1 was detected immunologically inside the nuclei showing a speckled appearance that colocalized with nucleoli (Fig. 5B, a and c).

### Efficient down-regulation of FOXA1 by *Foxa1* siRNA

**Figure 6:**
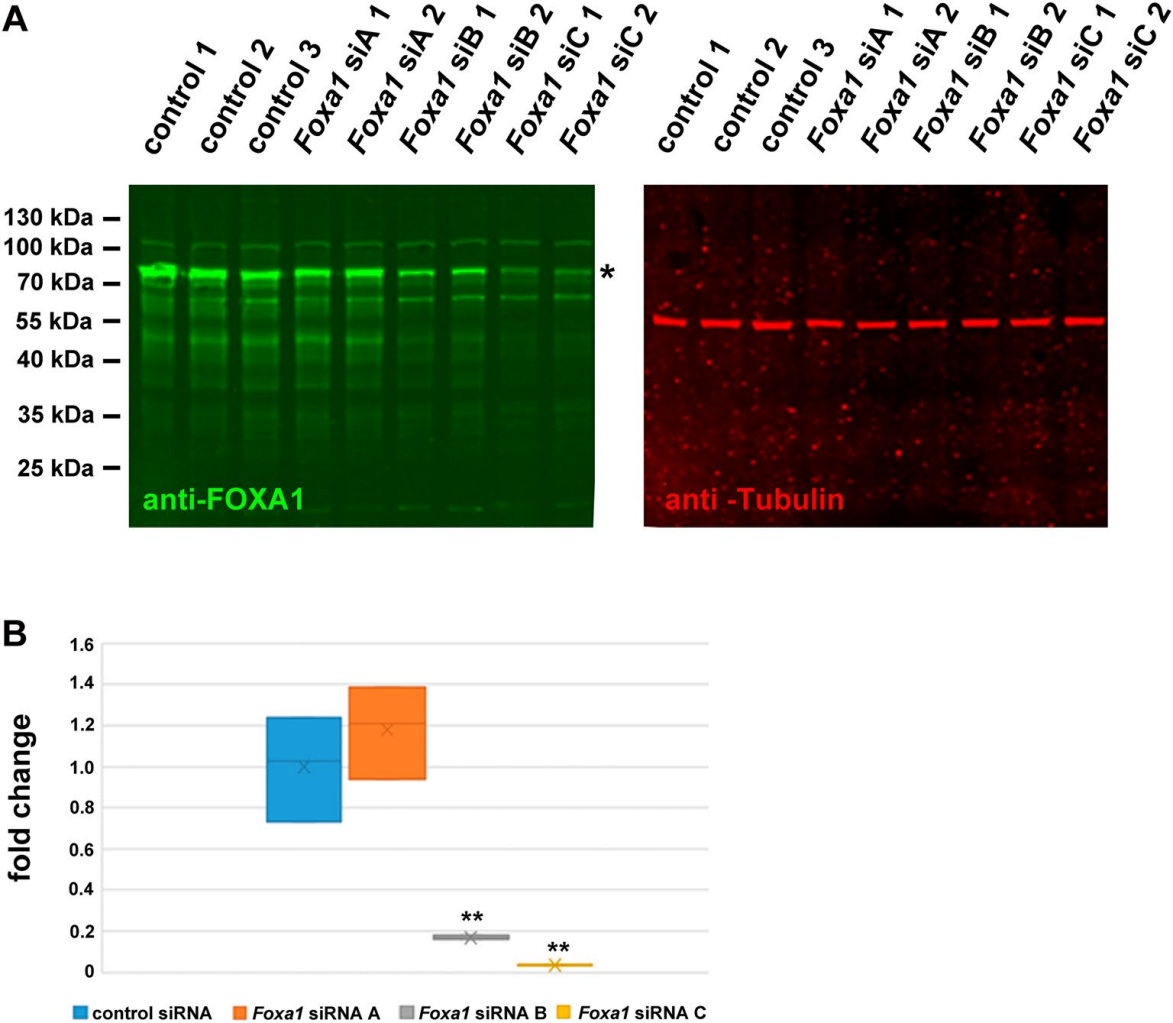
Knockdown of FOXA1::GFP by *Foxa1* siRNA duplexes. (**A**) Western blots demonstrating the reduction in FOXA1::GFP by *Foxa1* siRNA. *Foxa1::gfp* was co-transfected with either control siRNA (control) or one of the *Foxa1* siRNA duplexes A, B, or C (*Foxa1* siA, B, or C). 48 h post-transfection cells were harvested, and the cell lysates were analysed by Western blotting. Detection of FOXA1::GFP (green, ~ 77 kDa, asterisk) and α-Tubulin (red) on the same blot. The original blots are presented in Supplementary Figure S2. (**B**) Efficient reduction of FOXA1::GFP by *Foxa1* siRNA-mediated knockdown. The quantity of FOXA1::GFP was related to the quantity of α-Tubulin in the same lane and the fold changes in the relative quantities calculated to the average of the relative quantity in control siRNA transfected cells. Three biological replicates for each RNA duplex. Student’s T-test two-tailed, homoscedastic: siRNA A *p* = 0.412464, siRNA B *p* = 0.004772**, siRNA C *p* = 0.002733**.

We have demonstrated that *Foxa1* siRNA duplexes inhibit FOXA1-mediated activation of the *Odf2* promoter in reporter gene assays. To further prove that this effect is caused by siRNA-mediated knockdown of FOXA1, quantitative analyses were performed. Because of the low-level expression of *Foxa1*, we used FOXA1::GFP overexpression by co-transfection of *Foxa1::gfp* with either the control siRNA, or one out of the three *Foxa1* siRNA duplexes A, B, or C. Proteins were analysed by quantitative Western blotting using anti-FOXA1 antibodies, and anti-α-Tubulin antibodies (Fig. 6A, anti-FOXA1 in green, anti-α-Tubulin in red). Signal intensities of FOXA1::GFP at ~ 77 kDa (Fig. 6A asterisk) and α-Tubulin were quantified and their ratios calculated for each lane. The obtained relative intensities were related to the average relative intensity of the control siRNA samples giving the fold change of expression. We observed an efficient reduction of FOXA1::GFP by *Foxa1* siRNA duplexes B, or C, whereas no reduction was observed with the *Foxa1* siRNA A when compared to the control siRNA duplexes (Fig. 6B, *p* < 0.01 **).

### *Foxa1* siRNA-mediated knockdown affects the endogenous expression of *Foxa1*, *Odf2*/ODF2, and CP110

**Figure 7:**
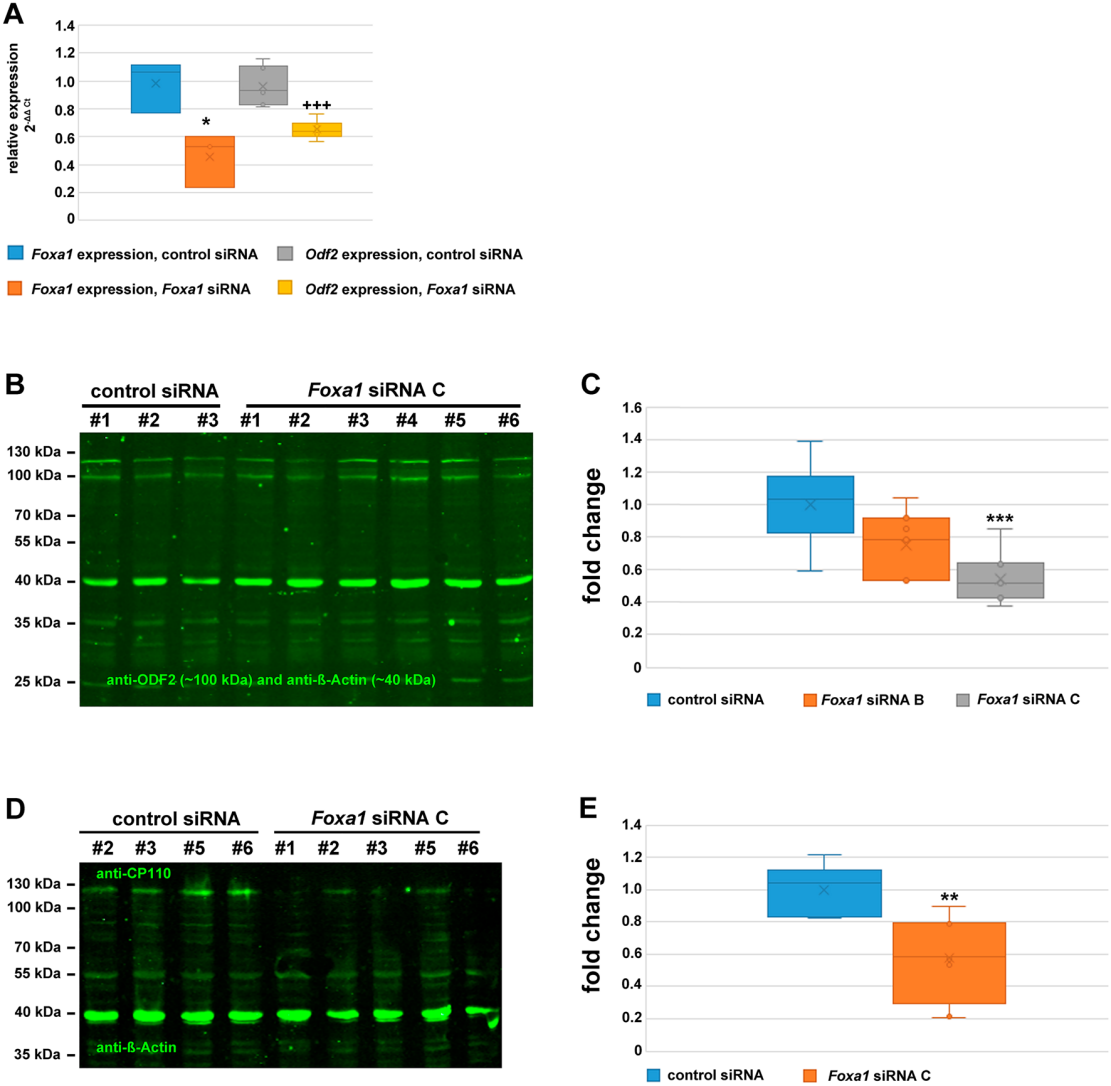
Decreased expression of *Foxa1*, *Odf2/*ODF2, *and* CP110 *by siRNA-mediated Foxa1 knockdown.* (**A**) Knockdown of *Foxa1* and *Odf2* transcripts by *Foxa1* siRNA. NIH3T3 cells were transfected with either the scrambled control siRNA (control siRNA) or the *Foxa1* siRNA (*Foxa1* siRNA) and transcription of *Foxa1* and *Odf2* quantified by RT-PCR. The relative expression of *Foxa1* or *Odf2* was calculated to both housekeeping genes, *Gapdh* and *Hprt*, by ΔCt. Their relative expression following siRNA-mediated depletion of *Foxa1* was compared to the control siRNA using the ΔΔCt-method and calculated as 2^-ΔΔCt^. Significant reduced expression of *Foxa1* (*p* = 0.02727533*) and *Odf2* (*p* = 0.00062325^+++^) related to the control siRNA. qRT-PCR was performed with at least three biological replicates, each measured in triplicate. Student’s T-test two-tailed, homoscedastic. (**B**) Western blot showing expression of ODF2 in NIH3T3 cells transfected with either the scrambled control siRNA (control siRNA, biological replicates #1 to #3) or the *Foxa1* siRNA C (biological replicates #1 to #6). Cells were harvested 48 h post-transfection and cultivation in serum-deprived medium. Detection of ODF2 (~ 100 kDa) and ß-Actin (~ 42 kDa) simultaneously on the same blot using the fluorescent-labeled secondary antibody anti-rabbit IgGCW800. An unspecific band was observed > 100 kDa. (**C**) *Foxa1* siRNA caused a reduction in ODF2 protein. The relative quantity of ODF2 was obtained by calculating the ratio between the quantity of ODF2 and ß-Actin in each lane, and relating the relative quantities to the average of the relative quantity obtained in control siRNA-transfected cells. Significant reduction of ODF2 expression by *Foxa1* siRNA C (*p* = 0.000631***). Six biological replicates each and a total of n loadings: control n = 7, siRNA B n = 7, siRNA C n = 9. (**D**) Western blot showing expression of CP110 in control siRNA, and *Foxa1* siRNA C transfected cells. (control siRNA: 4 biological replicates, *Foxa1* siRNA: 5 biological replicates). CP110 (< 130 kDa) and ß-Actin (~ 40 kDa) were both detected simultaneously on the same blot (in green). (**E**) FOXA1 knockdown caused a decreased quantity of CP110 (*p* = 0.002232**). The relative quantity of CP110 was calculated as described for ODF2 quantification using the quantity of ß-Actin as internal standard. For quantification six biological replicates for both, the control and siRNA were used and a total of n loadings were quantified: control n = 7, siRNA n = 8. Always Student’s T-test two-tailed, homoscedastic. The original blots are presented in Supplementary Figure S2.

Having proven an efficient reduction of FOXA1 protein by *Foxa1* siRNA-mediated knockdown, we asked whether knockdown of *Foxa1* could also be detected at the endogenous transcript level and whether a knockdown of FOXA1 would be accompanied by a reduction of *Odf2* transcripts and also of ODF2 proteins. NIH3T3 cells were, thus, transfected with the *Foxa1* siRNA, and transcripts of *Foxa1* and *Odf2* were quantified by qRT-PCR. We observed a reduction of *Foxa1* transcripts in *Foxa1* siRNA-transfected cells to ~ 0.5 × of that in control siRNA-transfected cells (Fig. 7A, p* 0.02727533). Furthermore, the reduction of *Foxa1* transcripts was accompanied by a reduction of *Odf2* transcripts to ~ 0.6 × compared to control siRNA-transfected cells (Fig. 7A; p*** 0.00062325). These results, therefore, indicate that transcription of *Odf2* is under control of FOXA1.

Finally, we quantified ODF2 proteins in lysates obtained from cells transfected with either the control siRNA or one of the *Foxa1* siRNA duplexes. We observed a significant reduction of ODF2 to approximately 0.6 × when FOXA1 was knocked down by *Foxa1* siRNA C (*p* = 0.000631***) (Fig. 7B, C; p***). Additionally, FOXA1 knockdown caused also a significant reduction of CP110, which has been annotated as a target gene of FOXA1^38^ (Fig. 7D, E  *p* = 0.002232**).

### FOXA1 is necessary for primary cilia formation

**Figure 8:**
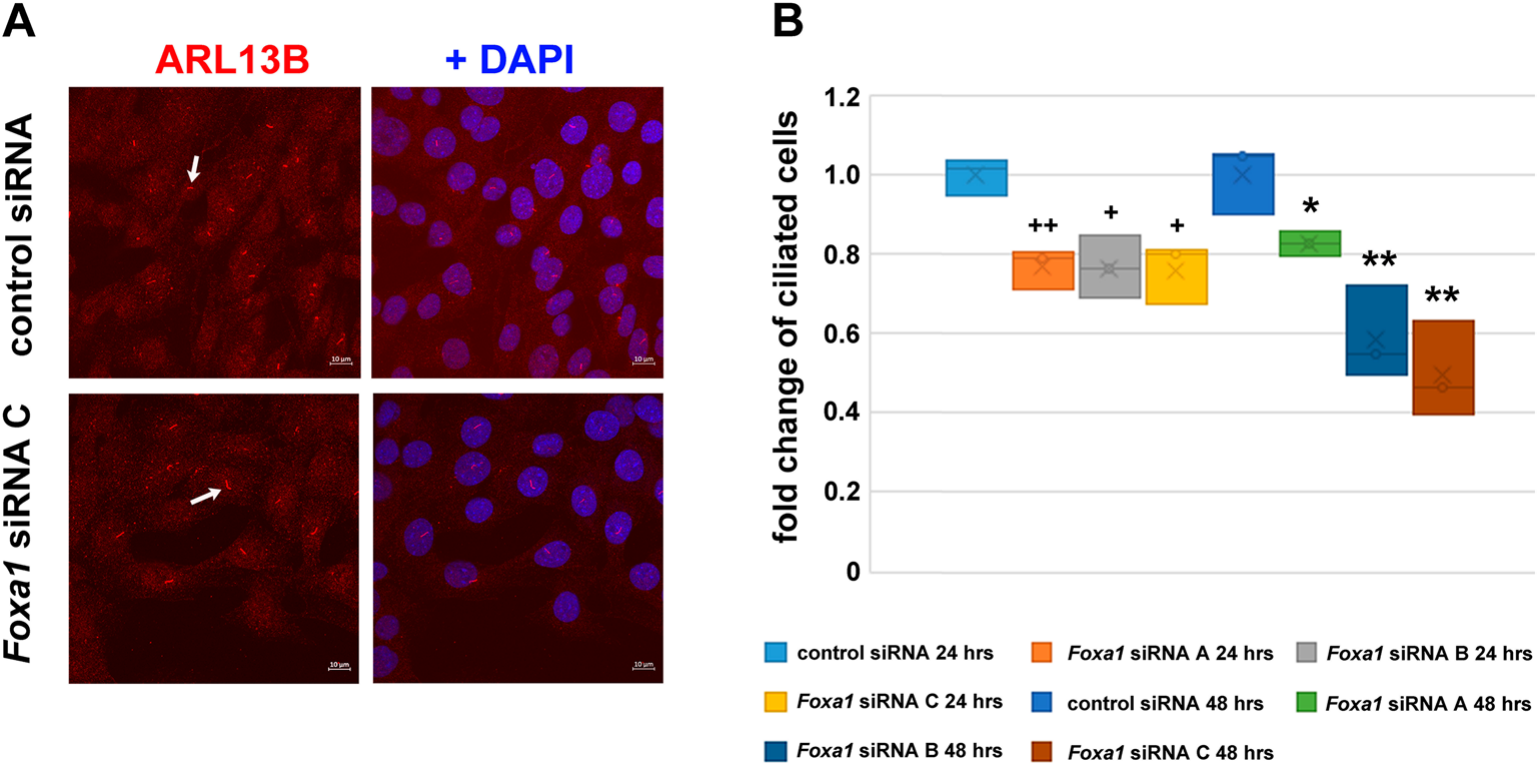
FOXA1 is mandatory for primary cilia formation. (**A**) Detection of primary cilia by immunodecoration of ARL13B (in red, arrows point to primary cilia). Merged images including nuclear counterstain with DAPI (in blue). Bars are of 10 µm. (**B**) siRNA-mediated depletion of *Foxa1* led to a decrease in ARL13B-positive primary cilia. NIH3T3 cells were transfected either with the scrambled negative control siRNA, or one of either *Foxa1* siRNA duplexes A, B, or C. 24 h post-transfection the medium was exchanged for serum starvation medium for cilia induction. Cells were fixed either after 24 h or 48 h in serum starvation medium and ARL13B decorated immunologically. ARL13B-positive primary cilia were manually counted. All experiments were performed in triplicates and n cells were counted: *Foxa1* siRNA A (24 h) n = 1,599, *FoxA1* siRNA B (24 h) n = 1,544, *FoxA1* siRNA C (24 h) n = 1,564, control (24 h) n = 1,593, *FoxA1* siRNA A (48 h) n = 1,593, *FoxA1* siRNA B (48 h) n = 1,540, *FoxA1* siRNA C (48 h) n = 1,590, control (48 h) n = 1,583. Student’s T-test two-tailed, homoscedastic, to control 24 h (^**+**^, siRNA A *p* = 0.004569, siRNA B *p* = 0.011402, siRNA C *p* = 0.010086), or to control 48 h (*, siRNA A *p* = 0.031788, siRNA B *p* = 0.008451, siRNA C *p* = 0.004429). *p* < 0.05 *, *p* < 0.01 **.

Since the amount of ODF2 is crucial for primary cilia generation we asked whether FOXA1 is involved in cilia formation. NIH3T3 cells were transfected with either one of the three different siRNA duplexes (A, B, or C) or the scrambled negative control siRNA duplex. 24 h post-transfection the medium was exchanged for serum starvation medium to induce cilia formation, and cells were cultivated for another 24 h or 48 h. Primary cilia were then immunologically decorated for ARL13B and manually counted (Fig. 8A). siRNA-mediated knockdown of FOXA1 caused a reduction of primary cilia to ~ 0.8 × when cultivated in serum starvation medium for 24 h (*p* < 0.05 ^+^ or *p* < 0.01^++^). Cultivation in serum starvation medium for 48 h caused a reduction of primary cilia to ~ 0.8 × by *Foxa1* siRNA A (*p* < 0.05*) and ~ 0.5 × in *Foxa1* siRNA B or C transfected cells (both *p* < 0.01**) (Fig. 8B). The *Foxa1* siRNA A has been turned out once more to be the least effective thus corroborating the data of the reporter gene assays (Fig. 3) and the Western blots (Fig. 6). However, it has to be kept in mind that the siRNA-mediated knockdown effects in reporter gene assays and Western blots were based on the reduction of the co-expressed *Foxa1*-plasmid which most likely accounts for the somehow differing results. Our results thus indicate that FOXA1 is mandatory for the formation of primary cilia.

### Co-immune precipitation revealed no direct interaction between FOXA1 and cJUN

**Figure 9:**
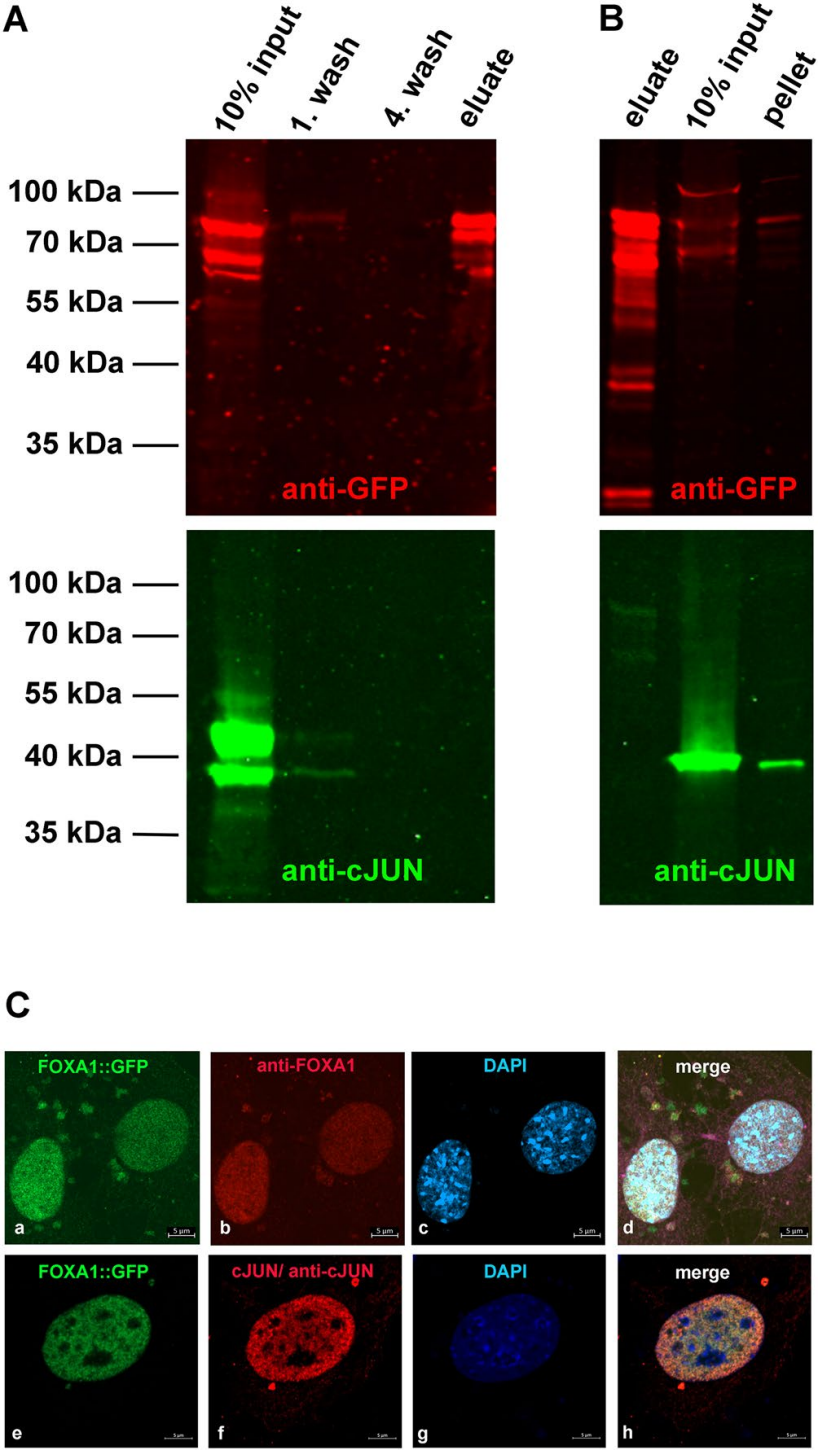
Interaction between FOXA1 and cJUN. (**A**, **B**) Co-immune precipitation of FOXA::GFP did not reveal direct interaction with cJUN. Cells were transfected with expression plasmids encoding *Foxa1::gfp*, and *Mekk1* (**A**) or *Foxa1::gfp* solely (**B**), and FOXA1::GFP captured by immobilized anti-GFP antibodies. Bead-bound proteins were eluted by boiling in SDS-sample buffer and the eluate, and aliquots of the input, the insoluble pellet, and the wash solutions were separated on denaturing SDS-gels. FOXA1::GFP was detected with anti-GFP antibodies (in red) and cJUN with anti-cJUN antibodies (in green). The unphosphorylated cJUN of ~ 39 kDa is present in A and B, whereas the phosphorylated cJUN of ~ 43 kDa was detected only when *Mekk1* was co-transfected (in **A**). Although the endogenous cJUN was detectable in the input fractions it did not co-precipitate with FOXA1::GFP (eluate). The original blots are presented in Supplementary Figure S2. (**C**) Co-localization of FOXA1::GFP and cJUN in transfected NIH3T3 cells. Antibody specificity of the anti-FOXA1 antibody was proven by expression of FOXA1::GFP (a, in green) and anti-FOXA1 antibody decoration (**b**, in red). Co-transfection of *cJun* with *Foxa1::gfp* followed by anti-cJUN antibody decoration (**f**, in red) demonstrated overlapping FOXA1::GFP fluorescence (**e**, in green) with anti-cJUN decoration (**h**, merged image). Nuclear staining (**c**, **g**, in blue) and merged images (**d**, **h**). Scales bares of 5 µm.

Our data demonstrated that FOXA1 is a transcriptional activator of *Odf2.* Furthermore, reporter gene assays suggested a positive interaction between FOXA1 and cJUN. To verify, we transfected cells with expression plasmids either *Foxa1::gfp* or *Foxa1::gfp* and *Mekk1,* followed by capturing of FOXA1::GFP using immobilized anti-GFP antibodies. Although FOXA1::GFP (of ~ 77 kDa) was successfully captured, demonstrated by its presence in the eluate of the bead-bound fraction, neither cJUN nor MEKK1-phosphorylated cJUN of either 36–39 kDa or 42–45 kDa, respectively, were co-precipitated (Fig. 9A). Thus, neither the unphosphorylated (Fig. 9A, B) nor the phosphorylated cJUN (Fig. 9A) were found to directly interact with FOXA1, despite FOXA1::GFP and cJUN colocalised in the nuclei of NIH3T3 cells (Fig. 9C).

## Discussion

Primary cilia are essential sensory organelles present on nearly all cells of the body. They are built in a cell cycle-dependent manner and are mainly found in quiescent cells. Generation of primary cilia depends on a crucial amount of ODF2/Cenexin, a basal body protein mandatory for cilia formation and viability^42–44^. Transcription of *Odf2* is cell cycle-dependent with upregulation in serum-starved cells and thus correlated with primary cilia formation^47^. We have previously identified TFs controlling the transcription of *Odf2*^47,50^. However, although few TFs have been identified to be involved in primary ciliation, as RFX3, the TF network regulating the formation of primary cilia has still to be figured out^51^. Motile ciliogenesis is controlled by the master regulator FOXJ1 in cooperation with RFX factors^35,52,53^. *Odf2* was not annotated as a direct target gene of FOXJ1 and neither RFX3 nor FOXJ1 TFs activate transcription of *Odf2* in NIH3T3 cells^37,47^. However, *Odf2* was down-regulated in RFX3-deficient ependymal cells of the mouse and possesses RFX3-binding sites identified by ChIP indicating that expression of *Odf2* is regulated by RFX3 in multi-ciliogenesis^54^. Furthermore, opposed to multiciliogenesis primary ciliogenesis is FOXJ1-independent^55^.

FOXJ1 belongs to the large family of evolutionarily conserved forkhead-box (Fox) TFs that have diverse functions in development and differentiation. FOX TFs are characterised by the conserved DNA-binding domain of ~ 110aa, denoted as fork head domain, first identified in FKH and the rat hepatocyte-enriched transcription factor HNF-3A^26,27^. Currently, more than 44 genes were annotated in both, mice and humans, and categorized into subclasses A to S. FOX proteins are essential TFs as the deletion of just one *Fox* gene very often leads to lethality, and mutations in *Fox* genes are associated with developmental disorders or diseases^31,56^. FOXJ1, alias HFH4, is the master regulator for motile cilia formation and is, therefore, essential for the execution of the specialized functions of epithelial cells harbouring motile cilia^32,33^.

The FOXO-proteins and FOXM1 function in cell cycle control. FOXM1 is a key regulator of both the G1/S phase and G2/M phase transition and is essential for proper mitotic progression^57^. Furthermore, FOXM1 is a component of the DREAM complex that inhibits transcription of target genes for cell proliferation in the quiescent state but promotes expression during the cell cycle^58^. The DREAM complex contacts DNA via the cell cycle genes homology region (CHR), which most commonly comprises the sequence TTTGAA^59^. The FOXO proteins are inhibitors of the cell cycle and regulate metabolism and lifespan. Like FOXM1 their activity is controlled by post-translational modifications. AKT-mediated phosphorylation of FOXO proteins caused their inactivation by translocation into the cytoplasm where they are stabilized by interaction with 14-3-3 scaffolding proteins^20^. FOXO proteins regulate cell proliferation via up-regulation of the cell cycle inhibitor p27^KIP1^ and downregulation of cyclin D1^60,61^. Their function as cell cycle inhibitors suggested a causal relationship to the transcriptional regulation of primary cilia formation, which are mainly found in quiescent cells. As a candidate approach, we focused on the sub-distal appendage protein and marker of the mother centriole and the basal body, ODF2/Cenexin, because it is mandatory for cilia formation and transcriptionally upregulated in serum-starved, cell-cycle arrested cells^42–44,46,50^. A consensus binding sequence for the forkhead-box TFs FOXO and FOXA1  was identified in the promoter region of the mouse *Odf2*-gene^48,49^. However, neither FOXO1  or its constitutively active form FOXO1ADA, nor FOXO3A activated transcription of *Odf2* in reporter gene assays. These data indicated that FOXO TFs are most likely not transcriptional activators of *Odf2* and largely rule them out as regulators of ciliation. A knockdown will therefore most likely have no effect on cilia formation. Instead, we found significant transcriptional activation of *Odf2* by FOXA1 and therefore focused on this TF.

The mammalian FOXA TFs were first identified in the rat liver and hence named hepatocyte nuclear factor 3 (HNF3) α, β, and γ, respectively FOXA1, 2, and 3^62^. Expression of FOXA1, FOXA2, and FOXA3 during development and in adult tissues exhibit overlapping but also distinct patterns^31^. Furthermore, FOXA1 is more widely expressed in adult tissues than FOXA2^31^. *Foxa1*-deficient mice survive until after birth but die between P2 (postnatal day 2) and P12 due to hypoglycemia and defects in kidney function^63,64^. FOXA2 is essential for node and notochord formation causing defects in the dorsal–ventral patterning of the neural tube and embryonic lethality when absent^65,66^. FOXA1 and FOXA2 regulate positively and negatively Sonic hedgehog (Shh)-signalling, via transcriptional regulation of the downstream effector *Gli2*, to specify the ventral midbrain progenitor identity^67^. FOXA3 is specifically expressed in the testis, in Leydig cells, and spermatids, and FOXA3-deficiency affected male fertility in mice^68^. Deletion of either one of these FOXA-factors in mice revealed that FOXA1 and FOXA2 can compensate for each other and that even FOXA3 may compensate for the deletion of both, FOXA1 and FOXA2, in late gestation in mice^69^. The observed phenotypes of the knockout mice strongly remind of ciliopathies. However, whether cilia are indeed affected has not been investigated and might also be hampered by the lethality of the mutants.

The FOXA TFs enable the access of tissue-specific TFs to their binding site and have, therefore, been denominated as ‘pioneer factors’^69,70^. Furthermore, they bookmark temporarily shut-off genes for easy reactivation^71^. Their function as pioneer factors has been annotated to the structural similarity of the forkhead box to histones H1 and H5^72,73^. Our reporter gene assays indicated a cross-talk between FOXA1 and cJUN, especially MEKK1-activated cJUN, in the transcriptional activation of *Odf2.* However, we could not find a direct interaction between FOXA1 and cJUN. Thus, the binding of FOXA1 to the *Odf2* promoter might enable concurrent binding of cJUN or phosphorylated cJUN to increase the expression of *Odf2* corroborating its function as a pioneer factor.

**Figure 10:**
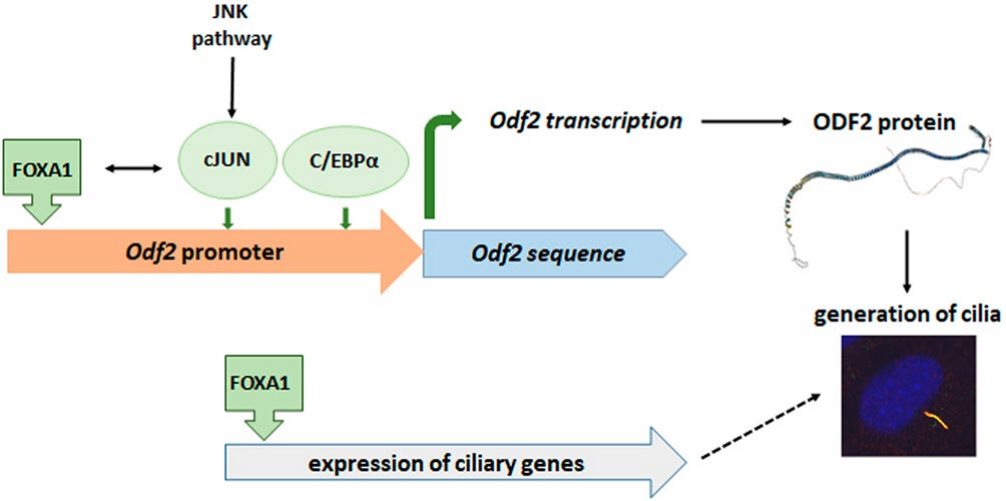
FOXA1 is a transcriptional activator of *Odf2,* which ultimately promotes cilia formation. FOXA1 binds to the sequence TGTTTAC at positions − 1768 to − 1775 of the *Odf2* promoter and promotes transcription of *Odf2*. The JNK-pathway via cJUN, and C/EBPα are also involved in the transcriptional activation of *Odf2*, although their binding sites have not been determined. Positive feedback was found between cJUN and FOXA1 in the transcriptional activation of the *Odf2* promoter, although a direct interaction could not be detected. According to the ChIP-seq dataset from the ENCODE Transcription Factor Targets Datasets, FOXA1 targets many more essential ciliary genes, including *CP110*, which we have shown is indeed regulated by FOXA1. The finally generated ODF2 protein, which is mandatory for the generation of primary cilia, eventually promotes cilia formation together with other ciliary proteins. The structure of the ODF2 protein (*Mus musculus)* was predicted by AlphaFold (AlphaFold DB version 2022-11-01, created with the AlphaFold Monomer v2.0 pipeline, licence CC-BY-4.0), and made available by EMBL-EBI (Wellcome Genome Campus, Hinxton, UK). The primary cilium was detected by immunological decoration of both, acetylated α-Tubulin (green) and ARL13B (red), shown here as merged image (yellow), and the nucleus stained with DAPI (blue) (inset).

To the known tasks of FOX TFs, our data add the novel function in the regulation of primary cilia formation (Fig. 10). We have shown that FOXA1 binds to its consensus sequence in the mouse *Odf2* promoter and activates the expression of *Odf2*/ODF2. Knockdown of FOXA1 not only downregulated *Odf2* transcripts and ODF2 proteins but also inhibited primary cilia formation. We found a significant reduction of *Foxa1* (to ~ 0.5x) as well as *Odf2* transcripts (to ~ 0.6x) after transfection of *Foxa1* siRNA that correlated with a 0.6-fold decrease in ODF2 proteins. Since *Foxa1* siRNA caused only a knockdown of FOXA1 but could not completely abolish it, FOXA1 is still present and, in addition, could also be compensated by FOXA2 that contributed to the continued expression of ODF2. *Foxa2* is also transcribed in NIH3T3 cells as was observed by nested RT-PCR (Supplementary Fig. S1) but due to a lack of validated siRNAs for the mouse FOXA2, its impact on primary ciliation could not be investigated. Reduced expression of ODF2 by FOXA1 knockdown provoked a decline of ciliated cells up to 0.55–0.75x (Fig. 8) compared to their reduction to 0.3–0.47x when *Odf2* was knocked down (all related to their respective controls) (Fig. 1). Our results, thus, demonstrate that *Odf2* is a target gene of FOXA1 that activates its transcription. Consequently, based on the availability of the ODF2 protein and further ciliary proteins, the generation of primary cilia is regulated by FOXA1. The ChIP-seq dataset from the ENCODE Transcription Factor Targets Datasets lists several ciliary genes as FOXA1 target genes, in between the ‘Gold Standard’ ciliary genes including *Odf2*, *CCP110/CP110*, *Cep290*, *Cep250*, *Cep164*, *Cep135*, and the Bardet-Biedl-Syndrome genes *BBS1*, *BBS4*, *BBS9*, *BBS10*, and *BBS12*, and many more ciliary genes^38,74,75^. These data indicate that FOXA1 is a TF for several ciliary genes and thus a regulator of ciliogenesis. To further corroborate, we demonstrated that CP110 is also affected by FOXA1 knockdown, which is, therefore, a FOXA1 target gene as indicated in the ChIP-seq dataset.

FOXA TFs are widely expressed during mouse embryogenesis and are required for the normal development of the nervous system and endoderm-derived organs. Their expression is even maintained in several adult tissues^64^. FOXA1 and FOXA2 TFs contribute to the maintenance of epithelial cell identity and their deregulated expression is associated with cancer formation^76,77^. FOXA1/2 are important for Sonic hedgehog (SHH) signalling by restricting the expression of the transcriptional mediators, *Gli1* and *Gli2*^67,78,79^. The hedgehog pathway is essential for vertebrate development, and aberrant activation of the pathway is associated with cancer formation^80^. Furthermore, the hedgehog signal transduction pathway is strictly dependent on the primary cilium^81^. The primary cilium is involved in both the promotion and inhibition of tumorigenesis, as has also been described for deregulated FOXA expression, but is generally absent in cancer cells^82,83^. The data, thus, indicated a link between FOXA1 TF, and hedgehog signalling, finally culminating on the primary cilium. Our data indicated that FOXA1 regulates primary ciliogenesis. We propose, therefore, that the deregulated expression of FOXA1 in cancer cells might affect the primary cilium, causing eventually aberrant hedgehog signalling.

## Methods

### Cell culture and immune-cytology

The mouse fibroblast line NIH3T3 was obtained from DSMZ (ACC59) and cultivated in Dulbecco’s Modified Eagle’s Medium (DMEM; GlutaMax™ with high glucose concentration (4.5 g/l); ThermoFisher Scientific, #10566), supplemented with 10% (v/v) fetal calf serum (FCS), 1000 U/ml penicillin and 1,000 µg/ml streptomycin at 37 °C and 5% CO_2_. Primary cilia were induced by cultivation in the above medium supplemented with 0.5% FCS (serum starvation medium) for 24 to 48 h.

For immune-cytology, cells were reseeded at a density of 2 × 10^5^ cells per well of a 6-well plate on glass coverslips. Cells were fixed in 3.7% paraformaldehyde (PFA) for 20 min at 4 °C, permeabilized with 0.3% Triton X-100 in PBS (phosphate-buffered saline) for 10 min at room temperature, followed by blocking non-specific binding sites by incubation in PBS containing 1% bovine serum albumin (BSA) and 0.5% Tween-20 for at least 1 h. Samples were incubated with the primary antibodies anti-acetylated α-Tubulin (clone 6-11B-1; Santa Cruz Biotechnology, Inc., #sc-23950, diluted 1:50), anti-ARL13B (Proteintech, #17711-1-AP, diluted 1:400), anti-FOXA1 (Proteintech 20411-1-AP, diluted 1:200), anti-ODF2 (ESAP15572, antibodies-online, diluted 1:100), anti-α-Tubulin (Oncogene, #Ab-1, diluted 1:100) at 4 °C overnight. Secondary antibodies used are goat anti-mouse-IgG-DyLight 488 (#35503, ThermoScientific), goat anti-mouse-IgG-AlexaFluor555 (#A21422, Lot 948498, Invitrogen/Mol. Probes), and goat anti-rabbit-MFP590 (#MFP-A1037, Molecular Probes, Eugene). DNA was counterstained with DAPI. Images were taken by confocal microscopy (LSM 980, Zeiss) and processed using Adobe Photoshop 7.0. Primary cilia were manually counted by visual inspection and scanning through all focal planes. Approximately 500 cells for each replicate were scored for the presence of a primary cilium. The total counts are given in the results as n, comprising all replicates.

### Transfection of cells

Plasmid DNA or siRNA was transfected using EndoFectin™ Max Transfection Reagent following the manufacturer´s instructions (GeneCopoeia #EF014). The mouse *Foxa1* gene (NM_008259) tagged with Myc-DDK in pCMV6-Entry was obtained from Origene (MR225487). The coding region of *Foxa1* was cloned in-frame to *Egfp* in *pEGFP-N1* (Clontech).

*Foxa1* knockdown was achieved using three unique 27mer siRNA duplexes (Origene, SR415184A, SR415184B, SR415184C, all used at a concentration of 10 nM), and a universal scrambled negative control siRNA duplex (Origene, SR30004; final concentration 25 nM).

For *Odf2* knockdown, *Odf2* siRNA (stealth siRNA ODF2MSS207236; Life Technologies; final concentration 40 nM) and control siRNA (siGenome Non-targeting siRNA #1; ThermoFisher Scientific Biosciences) were used. Additionally, the short hairpin constructs *sh3* (specifically targeting sequence gaactcctccaggagatac of mouse *Odf2/Cenexin*;^50^) or *K07* (Origene), which functions as control while lacking homology with any known mRNA, were used. For rescue, the expression plasmid encoding human Cenexin (*hCenexin*)^46^) was co-transfected, and to identify transfected cells, human histone *H4*^84^ fused to *egfp* was also co-transfected.

### Expression analyses by reverse-transcribed PCR (RT-PCR) and quantitative RT-PCR (qRT-PCR)

Total RNA was prepared using peqGOLD RNApure™ (PeqLab, Erlangen, Germany) following the recommendations of the manufacturer, and treated with Ambion® TURBO DNA-free™ DNase (Ambion #AM2238). The absence of genomic DNA was validated via PCR using amplification of *Gapdh* or *Hprt*, followed by cDNA synthesis (Maxima First Strand cDNA Synthesis Kit; ThermoFisher Scientific, #K1641). The following primers were used for expression analyses (RT-PCR): *Hprt* (mHPRT-for2 ggagtcctgttgatgttgcc/mHPRT-rev2 gggacgcagcaactgacatt), *Gapdh* (mGapdhf caccaccaactgcttagcc /mGapdhr cggatacattgggggtagg), *Foxa1* (for first PCR: Foxa1-for4 tattaccgccagaaccagc/ Foxa1-rev4 acgggtctggaatacacac (expected size: 793 bp), and for nested PCR: Foxa1-for1 ccttcaacgattgtttcgtc/Foxa1-rev2 gagaaggggtgattaaaggag (525 bp)), *Foxo1* (for first PCR: Foxo1-for1 gccctacttcaaggataaggg/Foxo1-rev1 cattgtggggaggagagtcag (842 bp) and for nested PCR: Foxo1-for2 caagagcggaaaatcaccc/Foxo1-rev2 ctggttcaatcctccgtaacttg (631 bp)), *Foxo3a* (for first PCR: Foxo3a-for1 cttcatgcgcgttcagaatg/Foxo3a-rev1 gtgtctggttgccgtagtg (752 bp) and for nested PCR: Foxo3a-for2 gtggatcatcaaccccgatg/Foxo3a-rev2 ccagcccatcattcagattc (449 bp)), and *Foxj1* (for first PCR: Foxj-for1 cattctcaacgccaaggc/Foxj-rev1 gatgctgtaggaaggatgtg (1027 bp) and for nested PCR: Foxj-for2 caacttctgctacttccgcc/Foxj-rev2 caagaaggtctcatcaaagtc (674 bp).

The quantitative real-time PCR (qRT-PCR) was performed on CFX96TM Real-Time System (Bio-Rad) using BlazeTaq SYBR Green qPCR mix 2.0 (GeneCopoeia, Rockville, MD). Primer efficiency was validated for all primer pairs and the specificity of the amplification reaction was verified by melting curve analyses. The following primer pairs were used for qRT-PCR: Foxa1_f3 (gacgccaagacattcaagcg)/Foxa1_r3 (atcgtgccaccttgacgaaa), Odf2_na-f4 (accatgaaggaccgctcttc)/Odf2 na-r4 (cgcacattcacagtgtcccc), and mHPRT-for2/mHPRT-rev2 and mGapdhf/mGapdhr as above. Three technical replicates were used for each analysis. The relative expression in each probe was calculated by ΔCt using the average Ct values of both housekeeping genes as reference. The relative expression in the experimental condition compared to the control was finally calculated as 2^−ΔΔCt^ using the ΔΔCt method. For the T-test, the averages of the relative expression of the technical replicates were used.

### Reporter gene assay

NIH3T3 cells were seeded at a density of 1 × 10^5^ cells per well of a 12 well plate. 24 h later cells were co-transfected with the reporter vector (1 µg/well), the internal control vector *phRL-SV40* (Promega, Madison, USA; 10–100 ng/well), and expression plasmids encoding transcription factors (each at 100 ng/well). As reporter vector either *2.2-pGL3* or one of the truncated *Odf2*-promoter vectors *(#1, 22.1, 7.6, 7.1, 1.5, A1, 0.5*) were used, in which part of the promoter region was cloned upstream of the firefly luciferase reporter *pGL3*^47^. The *Odf2* promoter constructs comprise the following promoter regions: *2.2* (− 1805/ + 358), *7.1* (− 1805/− 94), *7.6* (− 1805/− 1282), *22.1* (− 1805/− 1368), *#1* (− 1675/ + 1), *1.5* (− 797/ + 1), *A1* (− 797/ + 358), and *0.5* (− 94/ + 255)^47^. The following expression plasmids encoding transcription factors were used: *Foxa1* (Origene, MR225487), *Foxo1* (addgene 12148), *Foxo1 ADA* (addgene 12143, constitutively active, containing three point mutations Thr24Ala, Ser253Asp, Ser316Ala), *Foxo3a* (addgene 1787), *Foxj1* (Steven Brody, St. Louis), *Rfx3* (Walter Reith, Genf), *C/Ebpα* (addgene 12550), and *cJun* (*pFA2-cJun*, encoding the transactivation domain of aa 1–223) and *Mekk1* (MAP3K1; *pFC-MEKK*, aa 380–672) both from Stratagene (PathDetect cJun trans-Reporting System; Stratagene, La Jolla, USA).

To investigate the effect of FOXA1 knockdown on reporter gene activity, the *Foxa1* expression plasmid (Origene, MR225487) (100 ng/well), and either one of the *Foxa1* siRNA duplexes (Origene, SR415184A, SR415184B, or SR415184C, all used at a final concentration of 20 nM), or a universal scrambled negative control siRNA duplex (Origene, SR30004; final concentration 25 nM) were co-transfected. Cells were either cultivated in standard medium for 24 h post-transfection (cycling cells), or the medium was exchanged for serum starvation medium 24 h post-transfection, and cells cultivated for another 48 h (serum-starved cells). The Dual-Glo Luciferase Assay System (Promega, USA) was used for measuring firefly and Renilla luciferase activity using the Centro LB 960 luminometer (Berthold Technologies, Germany). Fold changes were calculated based on the relative luminescence (firefly luminescence/Renilla luminescence). Each experiment was performed in triplicates and repeated up to six times.

### Co-immune-precipitation and Western blotting

Cells were transfected with the expression plasmids either *Foxa1::Gfp* or *Foxa1::Gfp* and *Mekk1* (MAP3K1; *pFC-MEKK*, aa 380–672). 24 h post-transfection cells were trypsinised and lysed in RIPA-buffer (50 mM Tris pH 7.6, 150 mM NaCl, 1% Nonidet P40, 0.5% sodium desoxycholate, 0.1% SDS, protease inhibitor mix ProteoBlock Protease inhibitor Cocktail, Fermentas R1321) by vortexing and sonication (3 × 45 s each). The supernatant was incubated with GFP Trap Magnetic Agarose (chromotek, gtma-20) for 1 h at 4 °C on a rotating wheel. Beads were, thereafter, washed 4-times with 500 µl RIPA-buffer each, and 10% of each wash solution stored for later analysis. Bead-bound proteins were eluted by boiling in SDS-sample buffer containing ß-mercaptoethanol for 5 min.

Proteins were separated on denaturing SDS-gels and transferred to Hybond ECL. Blot membranes were blocked for 1 h in 5% dry milk in TBST (10 mM Tris–HCl pH 7.6, 150 mM NaCl, 0.05% Tween20), and incubated with the primary antibodies (rabbit anti-GFP, self-made, and mouse monoclonal anti-cJUN, proteintech 66313-1-Ig) overnight at 4 °C. For quantitative Western blots anti-ODF2 (ESAP15572, antibodies-online, diluted 1:1000), and anti-CP110 (Proteintech #12780-1-AP, diluted 1:3000) antibodies were used, and as the internal standard anti-ß-Actin antibody (proteintech #20536-1-AP). Primary antibodies were detected with the fluorescent-labelled secondary antibodies IRDye800CW goat anti-mouse IgG (LI-COR, #925-32210), and IRDye680RD goat anti-rabbit IgG (LI-COR, #925-68071), or with IRDye800CW goat anti-rabbit IgG (LI-COR, #925-32211) and IRDye680RD goat anti-mouse IgG (LI-COR, #925-68070). Images were captured with LI-COR Odyssey CLX and analysed using Image Studio Lite (LI-COR). Quantification was performed by calculating the ratio between the target protein and the internal standard (ß-Actin) in the same lane. The relative quantities were then related to the average relative quantity in the control, giving the fold change of target protein expression.

### Chromatin-immune-precipitation

NIH3T3 were transfected either with the *Foxa1* expression plasmid and the *Odf2* reporter vector *2.2-pGL3* or with *2.2-pGL3* exclusively to investigate the binding of the endogenous FOXA1. The chromatin-immune-precipitation (ChIP) protocol was a modification of Denissov et al.^85^. Briefly, cells were crosslinked with 1.1% formaldehyde in PBS (phosphate-buffered saline) containing 0.07 mM EDTA, 0.035 mM EGTA, and 3.5 mM Hepes for 30 min at room temperature, quenched by adding glycine to 0.125 M final concentration for 5 min at room temperature, and afterward washed twice with cold PBS. Cells were lysed in Hepes-buffer B (20 mM Hepes, 10 mM EDTA, 0.5 mM EGTA, 0.25% Triton X-100) for 10 min at 4 °C, followed by scraping, and cell collection by centrifugation. Cells were washed once in cold Hepes-buffer C (50 mM Hepes, 1 mM EDTA, 0.5 mM EGTA, 0.15 M NaCl), and finally resuspended in incubation-buffer (20 mM Hepes, 0.15 M NaCl, 1 mM EDTA, 0.5 mM EGTA, 1% Triton X-100 with proteinase inhibitor mix; ProteoBlock Protease inhibitor Cocktail, Fermentas R1321). Probes were sonified using 12 cycles (30 s on/30 s off) in the Bioruptor (Diagenode), and clarified by centrifugation.

Protein A Dynabeads (Dynal Biotech, #10001) were pre-cleared and pre-blocked before using for immune-precipitation. Beads were washed twice in incubation buffer, and once in incubation buffer containing 1 mg/ml BSA (bovine serum albumin), and incubated in incubation buffer containing 2 mg/ml BSA and 100 µg/ml sheared salmon sperm DNA overnight at 4 °C. The next day, beads were washed twice in incubation buffer containing 1 mg/ml BSA and incubated with the sheared chromatin for 1 h at 4 °C for pre-clearing. After magnetic beads collection, 10% of the supernatant was removed and stored as the input sample, and the remaining supernatant was transferred into new tubes and incubated with the antibody, either anti-FOXA1 antibody (2 µg/150 µl of chromatin; Proteintech #20411-1-AP) or rabbit IgG as control (4 µg/150 µl of chromatin; Diagenode #C154110206), together with fresh pre-cleared and pre-blocked beads at 4 °C overnight on a rotating wheel. The next day, beads were collected and washed twice in wash buffer 1 (20 mM Hepes, 1 mM EDTA, 0.5 mM EGTA, 0.15 M NaCl, 1% Triton X-100, 0.1% SDS, 0.1% sodium deoxycholate (NaDOC)), once in wash buffer 2 (20 mM Hepes, 1 mM EDTA, 0.5 mM EGTA, 0.5 M NaCl, 1% Triton X-100, 0.1% SDS, 0.1% NaDOC), once in wash buffer 3 (20 mM Hepes, 1 mM EDTA, 0.5 mM EGTA, 0.25 M LiCl, 0.5% NaDOC, 0.5% NP-40), and twice in wash-buffer 4 (20 mM Hepes, 0.5 mM EGTA, 1 mM EDTA). Beads and input samples were then incubated with 0.2 µg/µl RNase A in 10 mM Tris–HCl, pH 8.0, for 30 min at 37 °C followed by the addition of 20 µg Proteinase K and incubated in lysis buffer (50 mM Tris–HCl, pH 8.0, 10 mM EDTA, 1% SDS) at 65 °C overnight by constant agitation. DNA was extracted using NucleoSpin Gel and PCR Clean-up (Macherey–Nagel, #740609-250).

Binding site occupancy was investigated by qPCR using *Foxa1* primers flanking the consensus binding site in the *Odf2* promoter (Foxa1-7.6-for gaattctgagattatagctatg / Foxa1-7.6-rev gccttcagatgtatgtgtgc), or primers flanking a putative DREAM binding-site of sequence TTTGAA found at position + 27 to + 33 related to the transcription start (DREAM-E1-for ctcgtgacccagaagtgg / DREAM-E1-rev cggcagctcgcccattgg). Primers were validated first. The Ct-values obtained by either FOXA1- or control IgG-precipitation were adjusted to their respective input Ct (ΔCt), and the enrichment of the binding site sequence was calculated by adjustment to the control IgG (ΔΔCt).

### Statistical analyses

Data were processed and analysed using Excel. The box in the boxplots represents the 25–75th percentile. The median is given as a line, the mean by a cross. The whiskers show the minimum and maximum values inside the range given by Q1-1.5 × interquartile range (IQR) and Q3 + 1.5 × IQR. Data were analysed by Student’s T-test, *p* < 0.05*, *p* < 0.01**, *p* < 0.001***, *p* < 0.0001****.

## Supplementary Information

Supplementary Figure S1.

Supplementary Figure S2.

Supplementary Figure S3.

## Data Availability

There are no datasets generated during and/or analysed during the current study.

## References

[1] Hoyer-Fender S, Tucker KL, Caspary T (2013). Primary and motile cilia: their Ultrastructure and Ciliogenesis. Cilia and Nervous System Development and Function.

[2] Satir P, Christensen T (2007). Overview of structure and function of mammalian cilia. Annu. Rev. Physiol..

[3] Fisch C, Dupuis-Williams P (2011). Ultrastructure of cilia and flagella – back to the future!. Biol. Cell..

[4] Reiter J, Leroux MR (2017). Genes and molecular pathways underpinning ciliopathies. Nat. Rev. Mol. Cell Biol..

[5] Quarmby LM, Parker JDK (2005). Cilia and the cell cycle. J. Cell Biol..

[6] Kim SK, Tsiokas L (2011). Cilia and cell cycle re-entry. Cell Cycle.

[7] Plotnikova OV, Pugacheva EN, Golemis EA (2009). Primary cilia and the cell cycle. Methods Cell Biol..

[8] Wheatley DN (1971). Cilia in cell-cultured fibroblasts. III. Relationship between mitotic activity and cilium frequency in mouse 3T6 fibroblasts. J. Anat..

[9] Tucker RW, Pardee AB, Fujiwara K (1979). Centriole ciliation is related to quiescence and DNA synthesis in 3T3 cells. Cell.

[10] Schlereth K, Weichenhan D, Bauer T, Heumann T, Giannakouri E, Lipka D, Jaeger S, Schlesner M, Aloy P, Eils R, Plass C, Augustin HG (2018). The transcriptomic and epigenetic map of vascular quiescence in the continuous lung endothelium. eLife.

[11] Malumbres M, Harlow E, Hunt T, Hunter T, Lahti JM, Manning G, Morgan DO, Tsai LH, Wolgemuth DJ (2009). Cyclin-dependent kinases: a family portrait. Nat. Cell Biol..

[12] Lim S, Kaldis P (2013). Cdks, cyclins and CKIs: roles beyond cell cycle regulation. Development.

[13] Malumbres M (2014). Cyclin-dependent kinases. Genome Biol..

[14] Wang Z (2021). Regulation of cell cycle progression by growth factor-induced cell signaling. MDPI Cells.

[15] Johnson DG, Walker CL (1999). Cyclins and cell cycle checkpoints. Annu. Rev. Pharmacol. Toxicol..

[16] Sánchez I, Dynlacht BD (2005). New insights into cyclins, CDKs, and cell cycle control. Sem. Cell Dev. Biol..

[17] Carter ME, Brunet A (2007). FOXO transcription factors. Quick guide. Curr. Biol..

[18] Kops GJPL, Medema RH, Glassford J, Essers MAG, Dijkers PF, Coffer PJ, Lam EWF, Burgering BMT (2002). Control of cell cycle exit and entry by protein kinase B-regulated forkhead transcription factors. Mol. Cell Biol..

[19] Greer EL, Brunet A (2005). FOXO transcription factors at the interface between longevity and tumor suppression. Oncogene.

[20] Tzivion G, Dobson M, Ramakrishnan G (2011). FoxO transcription factors: regulation by AKT and 14-3-3 proteins. Biochim. Biophys. Acta..

[21] Kops GJPL, Dansen TB, Polderman PE, Saarloos I, Wirtz KWA, Coffer PJ, Huang TT, Bos JL, Medema RH, Burgering BMT (2002). Forkhead transcription factor FOXO3a protects quiescent cells from oxidative stress. Nature.

[22] Tothova Z, Kollipara R, Huntly BJ, Lee BH, Castrillon DH, Cullen DE, McDowell EP, Lazo-Kallanian S, Williams IR, Sears C, Armstrong SA, Passegué E, DePinho RA, Gilliland DG (2007). FoxOs are critical mediators of hematopoietic stem cell resistance to physiologic oxidative stress. Cell.

[23] Zhang Y, Xing Y, Zhang L, Mei Y, Yamamoto K, Mak TW, You H (2012). Regulation of cell cycle progression by forkhead transcription factor FOXO3 through its binding partner DNA replication factor Cdt1. Proc. Natl. Acad. Sci. USA.

[24] Liang R, Ghaffari S (2017). Mitochondria and FOXO3 in stem cell homeostasis, a window into hematopoietic stem cell fate determination. J. Bioenerg. Biomembr..

[25] Lai E, Prezioso VR, Smith E, Litvin O, Costa RH, Darnell JE (1990). HNF-3A, a hepatocyte-enriched transcription factor of novel structure is regulated transcriptionally. Genes Dev..

[26] Weigel D, Jäckle H (1990). The fork head Domain: A novel DNA binding motif of eukaryotic transcription factors?. Cell.

[27] Lai E, Prezioso VR, Tao W, Chen WS, Darnell JE (1991). Hepatocyte nuclear factor 3α belongs to a gene family in mammals that is homologous to the Drosophila homeotic gene fork head. Genes Dev..

[28] Jürgens G, Weigel D (1988). Terminal versus segmental development in the Drosophila embryo: the role of the homeotic gene fork head. Roux’s Arch. Dev. Biol..

[29] Weigel D, Jürgens G, Küttner F, Seifert E, Jäckle H (1989). The homeotic gene fork head encodes a nuclear protein and is expressed in the terminal regions of the Drosophila embryo. Cell.

[30] Hosaka T, Biggs W, Tieu D, Boyer AD, Varki NM, Cavenee WK, Arden KC (2004). Disruption of forkhead transcription factor (FOXO) family members in mice reveals their functional diversification. Proc. Natl. Acad. Sci. USA.

[31] Golson ML, Kaestner KH (2016). Fox transcription factors: from development to disease. Development.

[32] Chen J, Knowles HJ, Hebert JL, Hackett BP (1998). Mutation of the mouse hepatocyte nuclear factor/forkhead homolog 4 gene results in an absence of cilia and random left-right asymmetry. J. Clin. Invest..

[33] Brody SL, Yan XH, Wuerffel MK, Song SK, Shapiro SD (2000). Ciliogenesis and left-right axis defects in forkhead factor HFH4-null mice. Am. J. Respir. Cell Mol. Biol..

[34] Thomas J, Morlé L, Soulavie F, Laurençon A, Sagnol S, Durand B (2010). Transcriptional control of genes involved in ciliogenesis : a first step in making cilia. Biol. Cell..

[35] Choksi SP, Lauter G, Swoboda P, Roy S (2014). Switching on cilia: transcriptional networks regulating ciliogenesis. Development.

[36] Marshall CB, Mays DJ, Beeler JS, Rosenbluth JM, Boyd KL, Santos Guasch GL, Shaver TM, Tang LJ, Liu Q, Shyr Y, Venters BJ, Magnuson MA, Pietenpol JA (2016). P73 is required for multiciliogenesis and regulates the Foxj1-associated gene network. Cell Rep..

[37] Mukherjee I, Roy S, Chakrabarti S (2019). Identification of important effector proteins in the FOXJ1 transcriptional network associated with ciliogenesis and ciliary function. Front. Genet..

[38] Rouillard AD, Gundersen GW, Fernandez NF, Wang Z, Monteiro CD, McDermott MG, Ma’ayan A (2016). The harmonizome: a collection of processed datasets gathered to serve and mine knowledge about genes and proteins. Database (Oxford).

[39] Li JB, Gerdes JM, Haycraft CJ, Fan Y, Teslovich TM, May-Simera H, Li H, Blacque OE, Li L, Leitch CC, Lewis RA, Green JS, Parfrey PS, Leroux MR, Davidson WS, Beales PL, Guay-Woodford LM, Yoder BK, Stormo GD, Katsanis N, Dutcher SK (2004). Comparative genomics identifies a flagellar and basal body proteome that includes the BBS5 human disease gene. Cell.

[40] Pedersen LB, Veland IR, Schroder JM, Christensen ST (2008). Assembly of primary cilia. Dev. Dyn..

[41] Ishikawa H, Marshall WF (2011). Ciliogenesis: building the cell’s antenna. Nat. Rev. Mol. Cell Biol..

[42] Ishikawa H, Kubo A, Tsukita S, Tsukita S (2005). Odf2-deficient mother centrioles lack distal/subdistal appendages and the ability to generate primary cilia. Nat. Cell Biol..

[43] Salmon NA, Reijo Pera RA, Xu EY (2006). A gene trap knockout of the abundant sperm tail protein, outer dense fiber 2, results in preimplantation lethality. Genesis.

[44] Anderson CT, Stearns T (2009). Centriole age underlies asynchronous primary cilium growth in mammalian cells. Curr. Biol..

[45] Yang K, Tylkowski MA, Hüber D, Tapia Contreras C, Hoyer-Fender S (2018). ODF2/Cenexin maintains centrosome cohesion by restricting ß-catenin accumulation. J. Cell Sci..

[46] Soung NK, Kang YH, Kim K, Kamijo K, Yoon H, Seong YS, Kuo YL, Miki T, Kim SR, Kuriyama R, Giam CZ, Ahn CH, Lee KS (2006). Requirement of hCenexin for proper mitotic functions of polo-like kinase 1 at the centrosomes. Mol. Cell Biol..

[47] Pletz N, Medack A, Rieß EM, Yang K, Basir Kazerouni Z, Hüber D, Hoyer-Fender S (2013). Transcriptional activation of Odf2/Cenexin by cell cycle arrest and the stress activated signaling pathway (JNK pathway). Biochim. Biophys. Acta..

[48] Hedrick SM, Michelini RH, Doedens AL, Goldrath AW, Stone EL (2012). FOXO transcription factors throughout T cell biology. Nat. Rev. Immunol..

[49] Bochkis IM, Schug J, Ye DZ, Kurinna S, Stratton SA, Barton MC, Kaestner KH (2012). Genome-wide location analysis reveals distinct transcriptional circuitry by paralogous regulators FOXA1 and FOXA2. PLoS Genet..

[50] Tylkowski MA, Yang K, Hoyer-Fender S, Stoykova A (2014). Pax6 controls centriole maturation in cortical progenitors through Odf2. Cell Mol. Life Sci..

[51] Ait-Lounis A, Baas D, Barras E, Benadiba C, Charollais A, Nlend RN, Liègeois D, Meda P, Durand B, Reith W (2007). Novel function of the ciliogenic transcription factor RFX3 in development of the endocrine pancreas. Diabetes.

[52] You Y, Huang T, Richer EJ, Schmidt JE, Zabner J, Borok Z, Brody SL (2004). Role of f-box factor Foxj1 in differentiation of ciliated airway epithelial cells. Am. J. Physiol..

[53] El Zein L, Ait-Lounis A, Morlé L, Thomas J, Chhin B, Spassky N, Reith W, Durand B (2009). RFX3 governs growth and beating efficiency of motile cilia in mouse and controls genes involved in human ciliopathies. J. Cell Sci..

[54] Lemeille S, Paschaki M, Baas D, Morlé L, Duteyrat JL, Ait-Lounis A, Barras E, Soulavie F, Jerber J, Thomas J, Zhang Y, Holtzman MJ, Kistler WS, Reith W, Durand B (2020). Interplay of RFX transcription factors 1, 2 and 3 in motile ciliogenesis. Nucleic Acids Res..

[55] Jain R, Pan J, Driscoll JA, Wisner JW, Huang T, Gunsten SP, You Y, Brody SL (2010). Temporal relationship between primary and motile ciliogenesis in airway epithelial cells. Am. J. Respir. Cell Mol. Biol..

[56] Hannenhalli S, Kaestner KH (2009). The evolution of Fox genes and their role in development and disease. Nat. Rev. Genet..

[57] Laoukili J, Kooistra MRH, Brás A, Kauw J, Kerkhoven RM, Morrision A, Clevers H, Medema RH (2005). FoxM1 is required for execution of the mitotic program and chromosome stability. Nat. Cell Biol..

[58] Chen X, Muller GA, Quass M, Fischer M, Han N, Stutchbury B, Sharrocks AD, Engeland K (2013). The forkhead transcription factor FOXM1 controls cell cycle-dependent gene expression through an atypical chromatin binding mechanism. Mol. Cell Biol..

[59] Müller GA, Wintsche A, Stangner K, Prohaska SJ, Stadler PF, Engeland K (2014). The CHR site: definition and genome-wide identification of a cell cycle transcriptional element. Nucleic Acids Res..

[60] Medema RH, Kops GJ, Bos JL, Burgering BM (2000). AFX-like forkhead transcription factors mediate cell-cycle regulation by Ras and PKB through p27kip1. Nature.

[61] Schmidt M, Fernandez de Mattos S, van der Horst A, Klompmaker R, Kops GJPL, Lam EWF, Burgering BMT, Medema RH (2002). Cell cycle inhibition by FoxO forkhead transcription factors involves downregulation of cyclin D1. Mol. Cell Biol..

[62] Costa RH, Grayson DR, Darnell JE (1989). Multiple hepatocyte-enriched nuclear factors function in the regulation of transthyretin and alpha 1-antitrypsin genes. Mol. Cell Biol..

[63] Behr R, Brestelli J, Fulmer JT, Miyawaki N, Kleyman TR, Kaestner KH (2004). Mild nephrogenic diabetes insipidus caused by Foxa deficiency. J. Biol. Chem..

[64] Friedman JR, Kaestner KH (2006). The Foxa family of transcription factors in development and metabolism. Cell Mol. Life Sci..

[65] Weinstein DC, Altaba RIA, Chen WS, Hoodless P, Prezioso VR, Jessell TM, Darnell JE (1994). The winged-helix transcription factor HNF-3 beta is required for notochord development in the mouse embryo. Cell.

[66] Ang SL, Rossant J (1994). HNF-3ß is essential for node and notochord formation in mouse development. Cell.

[67] Mavromatakis YE, Lin W, Metzakopian E, Ferri ALM, Yan CH, Sasaki H, Whisett J, Ang SL (2011). Foxa1 and Foxa2 positively and negatively regulate Shh signalling to specify ventral midbrain progenitor identity. Mech. Dev..

[68] Behr R, Sackett SD, Bochkis IM, Le PP, Kaestner KH (2007). Impaired male fertility and atrophy of seminiferous tubules caused haploinsufficiency for Foxa3. Dev Biol..

[69] Iwafuchi-Doi M, Zaret KS (2016). Cell fate control by pioneer transcription factors. Development.

[70] Cirillo LA, Lin FR, Cuesta I, Friedman D, Jarnik M, Zaret KS (2002). Opening of compacted chromatin by early developmental transcription factors HNF3 (FoxA) and Gata-4. Mol. Cell..

[71] Caravaca JM, Donahue G, Becker JS, He X, Vinson C, Zaret KS (2013). Bookmarking by specific and nonspecific binding of Foxa1 pioneer factor to mitotic chromosomes. Genes Dev..

[72] Clark KL, Halay ED, Lai E, Burley SK (1993). Co-crystal structure of the HNF-3/fork head DNA-recognition motif resembles histone H5. Nature.

[73] Zaret KS, Caravaca JM, Tulin A, Sekiya T (2010). Nuclear mobility and mitotic chromosome binding: similarities between pioneer transcription factor FoxA and linker histone H1. Cold Spring Harb. Symp. Quant. Biol..

[74] van Dam TJP, Wheway G, Slaats GG, Huynen MA, Giles RH (2013). The SYSCILIA gold standard (SCGSv1) of known ciliary components and its applications within a systems biology consortium. Cilia.

[75] van Dam TJP (2019). CiliaCarta: an integrated and validated compendium of ciliary genes. PLoS One.

[76] Tang Y, Shu G, Yuan X, Jing N, Song J (2011). FOXA2 functions as a suppressor of tumor metastasis by inhibition of epithelial-to-mesenchymal transition in human lung cancers. Cell Res..

[77] Bernardo GM, Keri RA (2012). FOXA1: a transcription factor with parallel functions in development and cancer. Biosci. Rep..

[78] Epstein DJ, McMahon AP, Joyner AL (1999). Regionalization of Sonic hedgehog transcription along the anteroposterior axis of the mouse central nervous system is regulated by Hnf3-dependent and—independent mechanisms. Development.

[79] Filosa S, Rivera-Perez JA, Gomez AP, Gansmuller A, Sasaki H, Behringer RR, Ang SL (1997). Goosecoid and HNF-3beta genetically interact to regulate neural tube patterning during mouse embryogenesis. Development.

[80] Pasca di Magliano M, Hebrok M (2003). Hedgehog signalling in cancer formation and maintenance. Nat. Rev. Cancer..

[81] Bangs F, Anderson KV (2017). Primary cilia and mammalian hedgehog signaling. Cold Spring Harb. Perspect. Biol..

[82] Higgins M, Obaidi I, McMorrow T (2019). Primary cilia and their role in cancer. Oncol. Lett..

[83] Fabbri L, Bost F, Mazure NM (2019). Primary cilium in cancer hallmarks. Int. J. Mol. Sci..

[84] Albig W, Doenecke D (1997). The human histone gene cluster at the D6S105 locus. Hum. Genet..

[85] Denissov S, van Driel M, Voit R, Hekkelman M, Hulsen T, Hernandez N, Grummt I, Wehrens R, Stunnenberg H (2007). Identification of novel functional TBP-binding sites and general factor repertoires. EMBO J..

